# Transcriptomic Studies on Intracranial Aneurysms

**DOI:** 10.3390/genes14030613

**Published:** 2023-02-28

**Authors:** Rafal Morga, Joanna Pera

**Affiliations:** 1 Department of Neurosurgery and Neurotraumatology, University Hospital, ul. Jakubowskiego 2, 30-688 Krakow, Poland; 2 Department of Neurology, Jagiellonian University Medical College, ul. Botaniczna 3, 31-503 Krakow, Poland

**Keywords:** intracranial aneurysm, transcriptomic, RNA expression studies

## Abstract

Intracranial aneurysm (IA) is a relatively common vascular malformation of an intracranial artery. In most cases, its presence is asymptomatic, but IA rupture causing subarachnoid hemorrhage is a life-threating condition with very high mortality and disability rates. Despite intensive studies, molecular mechanisms underlying the pathophysiology of IA formation, growth, and rupture remain poorly understood. There are no specific biomarkers of IA presence or rupture. Analysis of expression of mRNA and other RNA types offers a deeper insight into IA pathobiology. Here, we present results of published human studies on IA-focused transcriptomics.

## 1. Introduction

The most common cause of spontaneous subarachnoid hemorrhage (SAH) is a rupture of an intracranial aneurysm (IA). This form of hemorrhagic stroke comprises about 5% of all strokes. Despite overall improvements in patients’ care, SAH is burdened with high mortality (approximately 50%) and disability rates—only 25% of patients who survived are likely to live independently. Most SAH patients have permanent neurological and cognitive deficits and remain dependent [1,2]. The prognosis is heavily influenced by the development of vasospasm and delayed cerebral ischemia (DCI). Vasospasm, which can be detected in approximately two thirds of SAH patients, may lead to DCI and subsequent neurological impairment. The incidence of spontaneous SAH is 8 persons in 100,000 person-years. The prevalence of unruptured IAs (UAs) in the general population is estimated at 3%. Most of the UAs remain unruptured since the risk of aneurysmal rupture is about 1% per year [3,4]. Unfortunately, it is still impossible to predict the fate of a particular IA. So far, only some risk factors of IA presence, growth, and rupture have been identified (for instance: female sex, hypertension, smoking, IA location, IA size), and based on them, a risk of an IA rupture can be estimated [4].

Molecular mechanisms underlying IA formation and rupture remain not fully recognized. Similarly, the knowledge about molecular drivers of systemic responses to the rupture of an IA is incomplete.

One of the approaches to investigate these aspects of IA pathobiology is to analyze alterations in RNA expression associated with the presence of IAs, their status (ruptured vs. unruptured), and sequels of SAH. The first studies focused on mRNA as a molecule containing the genetic information which is translated into proteins. However, over time, non-coding RNAs and RNA regulatory networks drew attention as well. Depending on the underlying question, RNA expression was analyzed in various samples, such as: IA wall, peripheral blood cells, and serum/plasma. The first broad gene expression profiling was performed by Peters et al. by means of the SAGE-Lite method in a single patient [5]. Afterwards, a microarray approach was used, subsequently replaced by RNA sequencing (RNAseq). In addition, by developing new bioinformatics tools, there is an increasing number of studies in which available original data are re-analyzed and/or existing datasets are combined.

In this review, we will focus on transcriptomics studies conducted on human-derived samples obtained from patients with IA. Firstly, a concise overview of original transcriptomics studies will be presented. Then, a brief summary of studies in which existing datasets were used (secondary studies) will be provided. A literature review was performed using PubMed and Web of Science. The search terms were “intracranial aneurysms”, “cerebral aneurysm”, “brain aneurysm” AND “gene expression”, and “RNA expression”. The identified reports were manually checked to select only transcriptomics studies on human-derived samples.

## 2. Original Studies

We identified 27 original studies which investigated RNA expression in the aneurysmal wall. Seven studies were focused on the mechanisms associated with IA rupture, eighteen on aneurysm formation, and in two reports alterations in RNA expression were analyzed both in present IAs and after their rupture. In studies on blood-derived samples, the corresponding numbers were the following: 24 studies, among them: 9 focused on the rupture-related changes (and complications of SAH in 2 studies), 10 on the IA presence, and in 5 studies, markers of IA formation and rupture were investigated.

### 2.1. Transcriptomics in IA Samples

These studies can be divided into two subgroups. The first one utilizes aneurysmal tissue and is focused on mechanisms involved in IA formation and rupture. IA samples and control arteries were obtained during neurosurgical procedures, except a study published by Weinsheimer et al. [6], where samples came from autopsies. In two other studies, expression data from available datasets served as controls [7,8]. In general, control vessels served as superficial temporal arteries [5,9,10,11,12,13,14,15,16,17,18,19,20,21,22,23] or middle meningeal arteries [11,23,24,25,26], and in single reports as arteriovenous malformation (AVM) feders [27] or cortical arteries [28]. One group did not specify which vessel was used as a control [29]. Numbers of analyzed samples vary from 3 [7,9,10] to 70 [26] per group. In two studies, in addition to vessels, peripheral blood samples were analyzed to investigate potential similarities between aneurysmal expression profiles and blood, searching for biomarkers of IA presence and/or rupture [22,30]. Three other studies comprised in vitro parts, which allowed to verify some findings from the expression analyses in vascular smooth-muscle cells [23,26] or endothelial cells’ cultures [31].

The first published analysis of global gene expression profiles in aneurysmal tissue was performed using the SAGE-Lite technique. Samples were obtained from a single patient—a 3-year-old girl with SAH: walls of a ruptured IA (RA) and a control vessel—the superficial temporal artery (STA). The analysis comprised 4924 and 3552 genes in the RA and STA samples, respectively, and revealed an overexpression of genes related to extracellular matrix, cell adhesion, and cell migration [5]. In subsequent studies, these two elements, i.e., differential expression of RNAs and their functional annotation, remained the core of the performed analyses. From non-coding RNAs, miRNAs were the most investigated class with or without a concomitant profiling of mRNAs [8,14,15,16,25,26]. In four studies, expression of lncRNAs was analyzed [16,17,20,30], and in two, circular RNA (circRNA) [22,31]. When mRNA expression was not directly measured, mRNA target prediction analysis was provided. Attempts to compare results of expression data on a single RNA molecule level are rather disappointing. For instance, Roder et al. in their meta-analysis of 5 microarray-based IA studies found that only 57 out of 507 reported differentially expressed genes (DEGs) were identified in more than 2 studies [32]. However, while looking at the functional annotations of differentially expressed RNAs, categories related to inflammatory reaction, immune system, cellular adhesion, extracellular matrix, muscles, apoptosis, and cellular signaling were identified as key players in the pathophysiology of IAs. Details are summarized in Table 1.

### 2.2. Transcriptomics in Blood-Derived Samples

In expression studies in blood samples, RNAs were isolated from whole blood [33,34,35,36,37], blood cells as a whole [38,39,40,41,42,43,44], or specifically from leukocytes [24], mononuclear cells [45,46,47], or neutrophils [48,49,50]. Circulating RNAs were isolated from plasma [51,52,53,54,55], serum [56], or circulating exosomes [57]. The main goals of this group of studies were: (i) search of biomarkers of IAs or their categories (RAs, UAs), and (ii) investigation of systemic consequences of IA rupture, including clinical status of SAH patients or SAH complications such as vasospasm [33,44] or DCI [38]. Circulating blood cells are notably sensitive to pathologic processes affecting the body. Only in one study was gene expression examined in intracranial, not peripheral, vessels—blood samples were obtained from IA lumen and IA proximal parent vessels [37]. The range of cohort sizes was from 3 [46] to 130 patients [24]. Korostynski et al. [41,42] and Morga et al. [43] analyzed differences in RNA expression profiles between acute and chronic phase of RA, whereas van’t Hof et al. searched for potential biomarkers of past aSAH (at least 2 years after RA) [40]. Similar to tissue-based studies, in most of the blood-based studies, mRNA expression was examined (cell-derived or circulating) [24,34,37,38,39,40,41,44,45,47,48,49,50,53]. However, non-coding RNAs were also studied—mainly miRNAs [33,42,51,52,54,55,56,57] and lncRNAs [24,35,53]. CircRNAs were investigated in two studies [36,46] and expression of different subtypes of small RNAs (piRNAs, rRNAs, tRNAs, snoRNAs, scRNAs) was presented in one report [43]. Functional analyses and target prediction for non-coding RNAs can be considered as standard approaches. In general, results of functional annotation resemble tissue-based studies. More details of this group of transcriptomics studies are presented in Table 2.

## 3. Studies Based on Existing Datasets

We identified 27 secondary studies which used datasets with RNA expression in the aneurysmal wall. Eighteen studies were focused on the mechanisms associated with IA rupture, twelve on aneurysm formation, and in nine, alterations in RNA expression were analyzed both in present IAs and after their rupture. In studies on datasets with blood-derived samples, the corresponding numbers were following: nine studies, among them: eight focused on the rupture-related changes, and one focused on the IA presence.

### 3.1. Transcriptomics in IA Samples

Along with the development of bioinformatic tools appeared a new type of study presenting re-analyzed data from available datasets, including expression data from the Gene Expression Omnibus (GEO). Approximately one third of these published secondary analyses utilized a single dataset [58,59,60,61,62,63,64,65,66] and two thirds leveraged data from two to eight datasets [67,68,69,70,71,72,73,74,75,76,77,78,79,80,81,82,83,84,85]. These studies did not provide any new additional clinical data but rather aimed to deepen the insight into molecular mechanisms of the IA pathophysiology by revealing key regulatory networks and interactions between investigated molecules. Although differential expression and functional annotation were examined, further analyses of co-expression networks with identification of hub RNA molecules, competing endogenous RNA (ceRNA) networks, or protein–protein interaction (PPI) networks became a standard approach. In some of these studies, specific areas of interest were predefined, such as: epithelial–mesenchymal transition [78], endoplasmic reticulum stress [81], immune environment [79,83], or ferroptosis [84,85]. In three studies, an attempt was made to identify potential therapeutic targets [71,82,83]. Sun et al. investigated expression profiles and networks in various aneurysms, including thoracic and abdominal aorta aneurysms [77]. More detailed information about this group of studies is provided in Table 3.

### 3.2. Transcriptomics in Blood-Derived Samples

Interestingly, the number of studies utilizing existing blood-based transcriptomics results is smaller than tissue-based studies. This could be explained by the availability of bio-samples. It is easier to design a new study and to obtain blood samples than aneurysmal specimens. In this category of studies, only three out of nine studies used data from at least two datasets [86,87,88], and six analyses were based on a single dataset [89,90,91,92,93,94]. Analytical methods used did not significantly differ when compared to tissue-based studies. Two reports comprised validation cohorts [87,88]. Table 4 shows more details.

## 4. Conclusions

**Figure 1:**
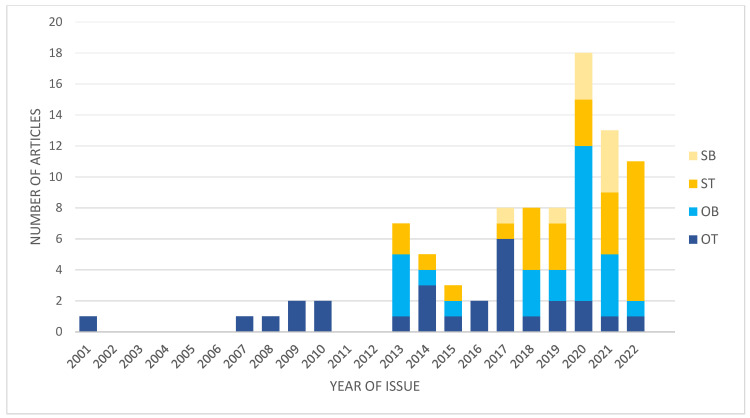
Graph presenting changes in numbers and types of studies focused on IA transcriptomics. OT, original studies using IA wall; OB, original studies using blood-derived samples; ST, secondary studies using tissue-derived data; SB, secondary studies using blood-derived data.

In the last decade, the number of studies focused on different aspects of transcriptomics in IAs significantly increased (Figure 1). This is associated with the technology development and bioinformatics allowing to analyze big data.

However, there are so many open questions regarding the pathophysiology of IAs and molecular mechanisms underlying the consequences of IA rupture. After more than 20 years of studies on the expression of coding and non-coding RNAs, it is obvious that there is not one single pathway responsible for IA formation or rupture. However, there are some networks, some groups of genes, that seem to play important role, such as immune/inflammatory response, extracellular matrix- or focal adhesion-related, cellular signaling, regulated cell death, and muscles. These terms are consistently repeated in presented studies, although in studies on blood-derived samples the most common identified pathways are those related to the immune/inflammatory response, cell death, or cellular metabolic processes. Secondary studies based on existing datasets echo these findings.

The existing expression studies are burdened with several limitations. These are human studies and not all factors that can affect gene expression are controllable and comparable between studied groups, including comorbidities, medications, and lifestyle habits. Next, time between sampling and placement of the sample on ice or transportation/storage solutions may impact expression measurements. Furthermore, the quality of the sample is important—what is the composition of the vessel/aneurysmal wall? For instance, there are acellular or hypocellular areas in some ruptured aneurysms. Moreover, the presence of even residual amounts of blood elements on the tissue will influence the results of expression analyses. Another important issue is the choice of the control tissue. In most studies, IAs and controls were obtained from different individuals. Some researchers used intracranial vessels (e.g., cortical arteries or AVM feders), whereas others used extracranial arteries. The anatomical differences between these vessels may affect the results of expression analyses. In 2019, Laarman et al. published results of their search for optimal controls in gene expression studies on IAs [95]. In blood-derived samples, a background cell count may play an important role for the analytical output. All these elements increase the heterogeneity of analyzed samples, including the RNA types. The secondary studies that use the existing datasets rarely pay much attention to clinical variables and focus on raw expression data.

With the progress of our knowledge about the gene expression, the regulatory mechanisms of transcription, and roles played by different classes of RNA, accompanied by the development of available research tools, researchers have started to analyze the alterations in other (not mRNA) types of RNA. However, it seems that we are still at the beginning of understanding the processes underlying the pathophysiology of IAs. Very little is known about the role of small noncoding RNAs other than microRNA. We do not even understand what the significance is of altered expression of gene isoforms. Further studies are needed to explain the role of gene expression and RNA molecules in the pathobiology of IAs and the consequences of their rupture. These studies cannot be limited to a pure transcriptomic analysis. Functional analyses using experimental approaches both in vitro and in vivo are needed to test the results from expression studies in a more complex environment of living cells or whole organisms.

## Figures and Tables

**Table 1:** Original studies on RNA expression in the intracranial aneurysm wall.

PMID/Reference	Cohorts	RNA Type	Detection/Verification Methods	Aim of the Study	Analytical Methods	Major Findings including Differentially Expressed RNAs, Involved Pathways/Functions (Top 5)
11283408 [5]	1 RA, 1 STA	mRNA	SAGE-Lite	gene expression profiling in RA	DEGs, putative function	overexpressed: fibronectin, *HLA-DR*, *MAC25*, *COL1A1*, *jun-B*; putative functions of DEGs: ECM constituent, MMP activation, ECM remodeling, collagen bridging, ECM
17878320 [6]	8 RA, 4 UA, 12 contralateral artery (postmortem)	mRNA	Affymetrix, Illumina microarray/qPCR	gene expression profiling in IA	DEGs, WebGestalt for functional annotation (KEGG), Cytoscape for interactions	810 IA candidate genes; KEGG: adherens junction, MAPK signaling pathway, focal adhesion, regulation of actin cytoskeleton, GnRH signaling pathway
18538937 [28]	6 RA, 4 UA, 4 AVM feder artery	mRNA	Agilent microarray/qPCR	gene expression profiling in IA, RA vs. UA	DEGs, IPA network, GO	521 DEGs; GO: antigen processing; IPA networks: MHC I and MHC II complex-related genes, antigen presentation
19752560 [9]	3 UA, 3 STA	mRNA	Affymetrix microarray/qPCR	gene expression profiling in UA	DEGs, DAVID for functional annotation (GO, KEGG)	1160 DEGs: 164 up, 996 down; GO-BP: cellular process, development, growth, regulation of biological process, reproduction; GO-CC: cell, envelope, extracellular region, membrane-enclosed lumen, organelle; GO-MF: binding, catalytic activity, enzyme regulator activity, signal transducer activity, transcription regulator activity; KEGG: focal adhesion, type 1 diabetes mellitus pathway, antigen processing and presentation pathway, complement and coagulation cascades
19228845 [10]	3 RA, 3UA, 3 STA	mRNA	Illumina microarray	gene expression profiling in IA	DEGs, functional annotation (GO, KEGG)	326 DEGs: 172 up (*KIAA1199*, *COL11A1*, *COL1A1*, *CDH2*, *POSTN*), 154 down (*C2orf40*, *CFD*, *CASQ2*, *RBPMS2*, *MUSTN1*); functional groups: collagens, cell communication, angiogenesis, inflammation, apoptosis; GO: organ and system development, cell–cell adhesion, actin cytoskeleton organization and biogenesis, actin binding, cytoskeletal protein binding; KEGG: focal adhesion, ECM–receptor interaction, cell communication
20044533 [24]	8 RA, 6 UA, 5 MMA	mRNA	Affymetrix microarray/qPCR	gene expression profiling in IA, RA vs. UA	DEGs, WebGestalt for functional annotation (GO), immunohistochemistry	159 DEGs: 131 common for RA and UA: 8 up, 123 down, 2 RA-specific (down: *CLSTN3*, *LIG1*), 26 UA-specific (up: *AIPL1, BLVRA*, *C18orf30*, *C2*, *C20orf59*); GO: **IA vs. ctrl/RA vs. UA**: up: Immune system process, Activation of plasma proteins during acute inflammatory response, Complement activation, Inflammatory response, Activation of immune response; **IA vs. ctrl**: down: Muscle contraction, Cell adhesion, Cell–matrix adhesion, Cell–substrate adhesion, Organ development
20487632 [11]	12 RA + 9 ctrl RA (MMA, STA), 10 UA + 12 ctrl UA (MMA, STA), 4 ctrl (STA, MMA)	mRNA	Affymetrix microarray/qPCR	gene expression profiling in IA and control vessels of IA patients and HC	DEGs, functional annotation	**RA vs. UA**: 10 up (*ELA2, MMP9, MMP14, ADAMTS1, CTSD*), 4 down (*TIMP3, TIPM4, BCL2L1, BCL2*); **ctrl RA vs. ctrl** UA: 1 up (*MMP14*), 2 down (*TIPM3, TIMP4*); **RA vs. ctrl**: 22 up (*ELA2, MMP2, MMP9, MMP12, MM14*), 8 down (*TIMP1, TIMP2, TIMP3, TIMP4, BCL2L1*); **ctrl RA vs. ctrl**: 6 up (*DAXX, FAS, MMP9, ADAMTS1, CTSD*), 6 down (*BCL2L1, TIMP3, TIMP4, LOX, COL1A2*); pathways: **RA vs. UA**: proteinases, inhibitors of proteinases, apoptosis, anti-apoptotic genes; **ctrl RA vs. ctrl UA**: proteinases, inhibitors of proteinases; **RA vs. ctrl**: proteinases, inhibitors of proteinases, apoptosis, anti-apoptotic genes, extracellular structural matrix proteins; ctrl RA vs. ctrl: proteinases, inhibitors of proteinases, apoptosis, anti-apoptotic genes, extracellular structural matrix proteins
24429729 [12]	15 IA, 17 STA	mRNA	Affymetrix microarray/qPCR	gene expression profiling in IA	DEGs	179 DEGs (up: *SPP1, IBSP, APOC1, OLR1, RGS1*; down: *PDE4C, AIF1L, TRPV1, CYP4B1, CXCL14*)
24938844 [13]	8 RA, 5 UA, 10 STA	mRNA	Agilent microarray/qPCR	gene expression profiling—signatures of RA	DEGs, DAVID for functional annotation (GO, KEGG)	**RA vs. UA**: 2047 DEGs: 430 up (*CSF3R, PFKFB4, FPR1, TFPI2, C19orf59*), 617 down (*COL10A1, EGR2, C20orf82, NOV, CPXM2*); functional analysis: GO up: Nucleosome, Defense response, Inflammatory response, Response to wounding, Immune response; KEGG up: Chemokine signaling pathway, Cytokine–cytokine receptor interaction, Fc γ R-mediated phagocytosis; GO down: Cell adhesion, Calcium ion binding, Extracellular matrix, Extracellular region part, Growth factor binding; KEGG down: N/A
27026628 [29]	22 RA, 21 UA, 16 cortical artery	mRNA	RNAseq/qPCR	comparison of gene expression profiles between RA, UA, control arteries	DEGs, Bioconductor for functional analysis (GO, KEGG)	**IA vs. ctrl**: 229 DEGs: 51 up (*COL10A1, CILP2, SFRP2, MEX3B, PTHLH*), 178 down (*FAM134B, SLC13A3, SERPIND1, GREB1, GJB6*); GO: inorganic anion transport, skeletal system development, regulation of developmental growth, plasma membrane region, ossification (predominantly: terms related to ECM and transmembrane transporter activity, blood vessel regulation); low-count genes expressed immunoglobulins; **RA vs. UA**: 1489 DEGs: 958 up (*MARCO, TGFBI, HPSE, CD300C, CD300E*), 531 down (*DOK6, CAMK2A, MYOZ3, IGHG4, TPH1*); GO: mitosis, positive regulation of cell development, negative regulation of G-protein-coupled receptor protein signaling pathway, cell–substrate adhesion (predominantly terms related to immune response, lysosomes, cell–cell interaction, in-cell regulation); KEGG: Lysosome, Osteoclast differentiation, Staphylococcus aureus infection, Phagosome, Leishmaniasis; low-count genes expressed: immunoglobulins
28057588 [7]	1 RA, 2 UA, 3 controls from GSE51878 (coronary artery SMC)	mRNA	RNAseq	gene expression profiling in IA and whole genome sequencing in additional cohort of 6 IA patients	DEGs, GeneCoDis3 for functional annotation (GO, KEGG), Cytoscape for PPI network	DEGs: 1459 up (*H19, PIK3R5, CHST15, A2M, SAMSN1*), 250 down (*HIST1H3J, FTH1P3, IFITM4P, ANXA2P1, ANXA2P3*); KEGG: Proteasome, Spliceosome, Huntington disease, Protein processing in endoplasmic reticulum, Parkinson disease; PPI network: 965 nodes (significant hub proteins: IKBKG, ACTB, MKI67IP)
27841008 [18]	6 RA, 6 UA, 12 STA	mRNA	Agilent microarray/qPCR	gene expression profiling in small RA (<10 mm) and larger UA (>10 mm)	DEGs, functional analysis with GO	**RA vs. UA**: 280 DEGs: 101 up, 179 down; GO: up: fever generation, cellular response to cycloheximide, heat generation, positive regulation of acute inflammatory response, regulation of organ formation; **RA vs. ctrl**: 2115 DEGs: 1007 up, 1108 down; GO: up: detection of molecule of bacterial origin, positive regulation of monocyte chemotaxis, T cell migration, regulation of monocyte chemotaxis, myeloid cell activation involved in immune response; **UA vs. ctrl**: 1910 DEGs: 755 up, 1155 down; GO: up: peptide antigen assembly with MHC protein complex, MHC protein complex assembly, T cell chemotaxis, T cell migration, neutrophil activation involved in immune response
28433851 [19]	7 RA, 20 UA, 20 STA	mRNA	Affymetrix microarray/qPCR	gene expression profiling in IA plus DNA methylation	DEGs, SAS system for functional annotation (GO, KEGG, BIOCARTA), DNA methylation analysis	2142 DEGs: 1203 up, 939 down; GO: multicellular organismal development, cell adhesion, regulation of transcription DNA-dependent, inflammatory response, cell differentiation; KEGG: cytokine–cytokine receptor interaction, PI3K-Akt signaling pathway, focal adhesion, signaling pathways regulating pluripotency of stem cells, TNF signaling pathway, proteoglycans in cancer; 11,022 differentially methylated sites: 6396 hyper, 4626 hypo; 14 genes as potentially associated with IA (*CXCL10, HK2, IL12RB1, IL21R, IL7R*)
29066233 [21]	15 IA, 17 STA	mRNA	Affymetrix microarray/qPCR	gene expression profiling in IA	DEGs, IPA pathways	179 DEGs; IPA pathways: communication between innate and adaptive immune cells, allograft rejection signaling, cytotoxic T lymphocyte-mediated apoptosis of target cells, graft vs. host disease signaling, antigen presentation pathway; TLR-2 signaling as a key player in IA formation
31316152 [23]	4 IA, 3 MMA/STA for RNAseq; 18 IA, 18 MMA/STA for qPCR	mRNA	RNAseq/qPCR	gene expression profiling in IA and selected protein expression in IA wall using immunostaining, culture of VSMC	DEGs, functional annotation (GO)	408 DEGs: 79 up (*KRT14, DAPL1, OACYLP, UBL4B, FFAR4*), 329 down *(HPSE2, ITLN1, CCL21, MYOC, ADIPOQ*); GO: up: immune response, cell adhesion, biological adhesion, defense response, inflammatory response; down: muscle contraction, muscle system process, striated muscle contraction, cell adhesion, biological adhesion; CCL3 as important chemoattractant for macrophages in IA
32355516 [30]	50 IA, 50 ctrl	mRNA	RNAseq/qPCR	gene expression profiling in IA and miR-566 and selected protein expression in IA	DEGs, miR-566 expression, western blot for protein expression	miR-566 up in IA; 256 DEGs: 12 up (*ALOX5, VEGF, CCR8, IGKC, PCAR*), 4 down (*VHL, ReIB, NIK, NGF2*)
24079748 [26]	14 RA, 14 MMA	mRNA, miRNA	Agilent microarray/qPCR	mRNA/miRNA profiling in RA	DEmRNAs, DEmiRNAs, IPA networks and pathways	30 DEmiRNAs: 1 up, 29 down (hsa-miR-140-3p, hsa-miR-7-1-3p, hsa-miR-29c-3p, hsa-miR-29c-5p, hsa-miR-23b-5p); 681 DEmRNAs as potential DEmiRNAs targets; IPA biological processes for target genes: migration of phagocytes, proliferation of mononuclear leukocytes, cell movement of smooth-muscle cells, differentiation of macrophages, stimulation of T lymphocytes
25868147 [27]	70 IA, 10 MMA	mRNA, miRNA	Agilent microarray/PCR	mRNA/miRNA expression profiling in IA, regulation of smooth-muscle contractility	DEGs, DEmiRNAs; DAVID and IPA for functional annotation (GO, networks); smooth-muscle cells’ cultures	1062 DEGs (*C1orf115, HLA-DRB1, FFAR4, SDK1, BRCA2*); 17 DEmiRNAs (hsa-miR-1274a, hsa-miR-135b-5p, hsa-miR-182-5p, hsa-miR-328, hsa-miR-337-3p); GO: regulation of muscle contraction, regulation of system process, regulation of smooth-muscle contraction, cell adhesion, biological adhesion; IPA networks—10 functional clusters; diseases and functions (Cellular Movement, Cellular Growth and Proliferation, Cardiovascular System Development and Function; Cell Morphology, Cancer, Organismal Injury and Abnormalities; Lipid Metabolism, Small Molecule Biochemistry, Molecular Transport; Cellular Growth and Proliferation, Cellular Movement, Cancer; Cardiovascular System Development and Function, Organ Morphology, Organismal Development)
26918470 [15]	7 UA, 10 STA	mRNA, miRNA	RNAseq for mRNA, Affymetrix microarray for miRNA	gene and miRNA expression profiling in UA	DEGs, DEmiRNAs, GOFAST for functional annotation (GO)	1028 DEGs: 623 up (*RP11-798K23.5, MMP13, SDS, MIR155HG, APOC1*), 405 down (*FNA20P, PLA2G2A, SFRP5, PCP4L1, PLIN1*); 1338 DEmiRNAs: up (miR-21-5p7, hsa-miR-1246, hsa-miR-6875-3p, hsa-miR-6753-3p, hsa-miR-4685-3p), down (hsa-miR-143-5p, hsa-miR-3195, hsa-miR-6068, hsa-miR-193b-5p, hsa-miR-6848-5p); GO: up: extracellular matrix, extracellular region part, proteinaceous extracellular matrix, extracellular region, cargo receptor activity; down: system process, galactosylceramide sulfotransferase activity, galactose 3-O-sulfotransferase activity, cytoskeletal protein binding, regulation of platelet-derived growth factor production; significant miR-mRNA pairs: miR-21—*PAIP2B*, miR-143—*COL1A1, COL5A1, COL5A2, MARCKS, TANC2*, miR-145—*ABCA1, ADAMTS2, BCAT1*
25300531 [14]	6 IA, 6 ctrl STA	miRNA	Agilent microarray/qPCR	miRNA profiling in IA	DEmiRNAs, DAVID and IPA for functional annotation (GO), Cytoscape for interaction networks	157 DEmiRNAs: 72 up (hsa-miR-298, hsa-miR-422a, hsa-miR-1299, hsa-miR-711, hsa-miR-1208), 85 down (hsa-miR-10b, hsa-miR-199b-5p, hsa-miR-1260, hsa-miR-139-5p, hsa-miR-143); functional analysis—DEmiRNAs-target mRNAs: Programmed cell death, Extracellular matrix organization, Response to oxidative stress, TGF-β signaling pathway, Smooth-muscle cell proliferation
34185228 [8]	29 RA, 20 controls from dataset GSE161870 (intercostal artery)	miRNA	Exiqon microarray/qPCR for miRNA and mRNA targets	miRNA expression profiling in aSAH patients	DEmiRNAs, DIANA to predict miRNA targets, functional annotation (GO, KEGG), TGFbeta pathway analysis; association with clinical status (aSAH severity, VSP)	70 DEmiRs: 67 down (hsa-miR-143-3p, hsa-miR-4328, hsa-miR-145-5p, hsa-miR-23c, hsa-miR-143-5p), 3 up (hsa-miR-642b-3p, hsa-miR-103a-2-5p, hsa-miR-4732-5p); KEGG for 10 top miRs: Fatty acid biosynthesis, Wnt signaling pathway, PI3K-Akt signaling pathway, ErbB signaling pathway, MAPK signaling pathway; GO-CC: cytoskeleton, intracellular, nucleus, cytoskeleton organization, cytoplasmic membrane-bounded periplasmic space; GO-MF: hydrolase activity, lipid binding, carbohydrate binding, receptor activity, phosphorus phosphatase activity; GO-BP: microtubule organization center, catabolic process, protein transport, cellular homeostasis, mitochondrion organization; decreased in patients with WFNS 3 and 4: miR-125b-5p, miR-143-3p; decreased in patients with VSP: miR-125b-5p, miR-143-3p
27751926 [16]	6 RA, 6 UA, 12 STA (the same patients)	mRNA, miRNA, lncRNA	Agilent microarray for mRNA and lncRNA, Affymetrix microarray for miRNA	RNAs expression profiling in IA, ceRNA regulatory network in IA	DEGs, DElncRNAs, DEmiRNAs, DAVID for functional annotation (GO, KEGG), MiRanda to predict miRNA targets, ceRNA score and network	286 DEmiRNAs: 234 up, 52 down; 1518 DElncRNAs: 413 up, 1105 down; 2545 DEGs: 1150 up, 1395 down; GO: cell adhesion, regulation of vascular smooth muscle, positive regulation of protein kinase activity, axon guidance, dorsal aorta morphogenesis; KEGG: axon guidance, cell adhesion molecules (CAMs), oxitocin signaling pathway, cGMP-PKG signaling pathway, vascular smooth-muscle contraction; 1461 miRNA–lncRNA interaction, 9269 miRNA–mRNA interactions; 8401 miRNA–lncRNA–mRNA interactions
27965470 [17]	12 RA, 15 UA, 27 STA	mRNA, lncRNA	Agilent microarray	mRNA and lncRNA expression profiling in IA	DEGs, DElncRNAs, DAVID for functional annotation (GO, KEGG)	2926 DEGs: 1511 up, 1415 down; 4129 DElncRNAs: 876 up, 3253 down; GO: up: immune response, inflammatory response, regulation of immune response, interferon-γ-mediated signaling pathway, innate immune response; down: muscle contraction, muscle organ development, positive regulation of glucose import, smooth-muscle contraction; KEGG: up: lysosome, phagosome, Staphylococcus aureus infection, tuberculosis, leishmaniasis; down: cGMP-PKG signaling pathway, vascular smooth-muscle contraction, proteoglycans in cancer, focal adhesion, regulation of lipolysis in adipocytes; lncRNA–mRNA networks represented in: immune response, inflammatory response, muscle contraction pathway
28009235 [20]	12 IA, 12 STA	mRNA, lncRNA	CapitalBio microarray/qPCR	mRNA and lncRNA expression profiling in IA	DEGs, DElncRNAs, GeneSpring, functional annotation (GO, KEGG)	2545 DEGs: 1150 up, 1395 down; 1518 DElncRNAs: 413 up, 1105 down; GO: up: T cell chemotaxis, T cell migration, lymphocyte chemotaxis, lymphocyte migration, regulation of lymphocyte apoptotic process; down: smooth-muscle contraction, muscle contraction, muscle system process, striated muscle cell differentiation, muscle cell differentiation; KEGG: up: chemokine signaling pathway, cell adhesion molecules, Toll-like receptor signaling pathway, lysosome, B-cell receptor signaling pathway; down: vascular smooth-muscle contraction, focal adhesion, dilated cardiomyopathy, adipocytokine signaling pathway, phosphatidylinositol signaling system; CCL5 targeted by 17 lncRNAs as a central player in IA pathogenesis
33023605 [25]	4 IA, 4 STA; for peripheral blood leukocytes 2 tiers: 130 IA, 130 HC	mRNA, lncRNA	RNAseq/qPCR	mRNA and lncRNA expression profiling in IA and validation of selected lncRNA expression in peripheral blood leukocytes	DEmRNAs, DElncRNAs, DAVID for functional annotation (GO, KEGG), Cytoscape for CNC network	1193 DElncRNAs: 900 up (LncRNA ENST00000508090, LncRNA ENST00000576153, LncRNA ENST00000569478, LncRNA ENST00000478738, LncRNA ENST00000463972), 293 down (LncRNA ENST00000446406, LncRNA ENST00000469162, LncRNA ENST00000469162, LncRNA ENST00000579688, LncRNA ENST00000474353); 2127 DEGs: 1297 up, 831 down; GO-BP up: defense response to virus, type I interferon signaling pathway, inflammatory response, neutrophil degranulation, innate immune response; GO-CC up: membrane, plasma membrane, Golgi membrane, phagocytic vesicle membrane, cytosol; GO-MF up: protein binding, tumor necrosis factor receptor binding, T cell receptor binding, receptor activity, MHC class I protein binding; GO-BP down: cell adhesion, SRP-dependent cotranslation, translation, translational initiation, nuclear-transcribed mRNA; GO-CC down: extracellular matrix, cytoskeleton, receptor complex, Z-disc, proteinaceous extracellular matrix; GO-MF down: structural constituent of ribosome, actin filament binding, Wnt-activated receptor activity, actin binding, heparin binding; KEGG: up: measles, natural killer cell-mediated signaling, T cell receptor signaling pathway, cytokine–cytokine receptor interaction, NOD-like receptor signaling; down: ribosome, adherens juction, regulation of lipolysis, dilated cardiomyopathy, axon guidance; 5 DElncRNAs in blood: lncRNA ENST00000471220, lncRNA ENST00000607042, lncRNA ENST00000478738, MALAT1, lncRNA ENST000000576153; good predictive value of lncRNA ENST00000607042 in IA
31254341 [22]	2 RA, 2 UA, 4 STA; for blood study: 24 RA, 6 UA, 30 HC	mRNA, circRNA	RNAseq/qPCR	mRNA and circRNA expression profiling in IA, ceRNA regulatory network in IA, expression of selected circRNA in peripheral blood	DEGs, DEcircRNA, functional annotation (GO, KEGG), circRNA–miRNA–mRNA network (TargetScan, miRanda, miRTarBase)	DEGs: 1297 up, 831 down; DEcircRNA/host genes: 116 up (chr17: 7480128–7480270: +/SNORD10, chr14: 23371395–23371591: −/RBM23, chr1: 66378927–66384518: +/PDE48, chr17: 80992910–81006661: −/B3GNTL1, chr19: 18285849–18286507: +/IF130), 199 down (chr11: 92085261–92088570: +/FAT3, chr2: 179542851–179542935: −/TTN, chr12: 56094682–56094938: −/ITGA7, chr2: 179515969–179516047: −/TTN, chr5: 38523520–38530768: −/LIFR); GO: up: Inflammatory response, Defense response to virus, Type I interferon, TNF receptor binding, T cell receptor binding; down: Cell adhesion, Extracellular matrix, Cytoskeleton, Ribosomal structure, Actin filament binding; KEGG: up: NK cell-mediated cytotoxicity, T cell receptor, Cytokine–cytokine receptor interaction, NOD-like receptor, Necroptosis; down: Ribosome, Adherens junction, Regulation of lipolysis in adipocytes, Axon guidance, Parkinson’s disease; hsa_circ_0072309 and hsa_circ_0008433 as potential IA biomarkers
34611229 [31]	18 RA, 16 UA	circRNA	Affymetrix microarray/qPCR	profiling of circRNA expression in EC from RA vs. UA and shear stress effect on circRNA and miRNA expression in EC culture	DEcircRNAs, in vitro analyses	RA vs. UA: 9 up (circRNA_0004543, circRNA_0079586, circRNA_0000231, circRNA_0003204, circRNA_0454542); 6 down (circRNA_0003492, circRNA_0011032, circRNA_0004264, circRNA_0002331, circRNA_0004528); MPO as a potential biomarker for IA rupture

aSAH, aneurysmal subarachnoid hemorrhage; AVM, arteriovenous malformation; BP, biological process; CC, cellular component; ceRNA, competing endogenous RNA; ctrl, control; DCI, delayed cerebral ischemia; DEcircRNAs, differentially expressed circRNA; DEGs, differentially expressed genes; DElncRNAs, differentially expressed lncRNA; DEmiRNAs, differentially expressed miRNAs; DEmRNAs, differentially expressed mRNAs; ECM, extracellular matrix; GO, Gene Ontology; HC, healthy control; IA, intracranial aneurysm; IPA, Ingenuity Pathway Analysis; KEGG, Kyoto Encyclopedia of Genes and Genomes; MF, molecular function; MMA, middle meningeal artery; PPI, protein–protein interaction; qPCR; quantitative PCR; RA, ruptured aneurysm; SMC, smooth-muscle cell; RNAseq, RNA sequencing; STA, superficial temporal artery; UA, unruptured aneurysm; VSMC, vascular smooth-muscle cell; VSP, vasospasm; WFNS, World Federation of Neurological Surgeons. In some studies, GO and KEGG terms were not analyzed separately for up- and down-regulated DERNAs but only for DERNAs as a whole. Data presented in the table reflect available data.

**Table 2:** Original studies on RNA expression in blood-derived samples.

PMID/Reference	Cohorts	Source	RNA Type	Detection/Verification Methods	Aim of the Study	Analytical Methods	Major Findings including Differentially Expressed RNAs, Involved Pathways/Functions (Top 5)
24135536 [38]	32 RA: 16 DCI+, 16 DCI−	peripheral blood cells	mRNA	RNG/MRC microarray/qPCR	Gene expression profiling in in aSAH w/wo DCI	DEGs	17 DEGs: 10 up in DCI+ (*NAMPT, NRG1, HGMCL, HTRA1, AF034187_186, PPP2R5C*), 7 up in DCI− (*EIF3K, HCST, PSMC3IP, TRPC4AP, SUSD3*)
23512133 [39]	43 RA, 18 ctrl	peripheral blood cells	mRNA	Illumina microarray/qPCR	Gene expression profiling in RA	DEGs, WebGestalt for functional annotation (GO, KEGG), cell type-specific gene expression (GSE28491)	135 DEGs: 78 up (*ACSL1, ALPL, ANKRD22, ANXA3, ARG1*), 57 down (*ABLIM1, ATP8B2, BCL11B, C2orf89, CCND2*); GO: all DEGs: immune system process, immune response, lymphocyte differentiation, leukocyte differentiation, T cell differentiation; up DEGs: defense response, innate immune response, negative regulation of cytokine production during immune response, immune response, pentose biosynthetic process; down DEGs: immune system process, immune response, lymphocyte differentiation, leukocyte activation, lymphocyte activation; KEGG: all DEGs: Hematopoietic cell lineage, Cytokine–cytokine receptor interaction, Primary immunodeficiency, T cell receptor signaling pathway, Systemic lupus erythematosus; up DEGs: Systemic lupus erythematosus, Metabolic pathways, Insulin signaling pathway, Fructose and mannose metabolism, Starch and sucrose metabolism; down DEGs: Hematopoietic cell lineage, Primary immunodeficiency, T cell receptor signaling pathway, Cytokine–cytokine receptor interaction, Cell adhesion molecules (CAMs); up: transcripts related to monocytes, neutrophils, down: transcripts related to T cell
24152840 [45]	15 RA, 15 UA, 15 ctrl	PBMC	mRNA	Agilent microarray	gene expression profiling in peripheral blood cells in IA	DEGs	DEGs: **RA vs. UA**: 1 up (*JUN*), 6 down (*SNCA, MMP1, IFI27, FN1, MMP9*); **UA vs. ctrl**: 14 up (*HNRNPA1, GBP1, ITGB2, STAT1, TP53*), 48 down (*E2F1, WIPF1, TUBA4A, CXCR4, LMNA*); **RA vs. ctrl**: 16 up (*ZFAT, ITGB2, SUMO1, C22orf9, SMA4*), 37 down (*PTGS2, ACTN1, GPR84, RAB32, PTX3*); functional gene groups: extracellular matrix structural proteins, heat shock proteins, cytoskeleton proteins, intracellular and extracellular signal cascade proteins, pro-apoptotic genes
26439625 [40]	119 RA, 118 ctrl (2/3 discovery, 1/3 replication)	peripheral blood cells	mRNA	Illumina microarray	Gene expression profiling in past aSAH (>2 years)	DEGs, WGCNA for co-expression network (co-differential co-expression, CDC; differential co-expression, CD), DAVID for functional annotation (GO)	No DEGs including previously identified in GWAS studies IA-associated genes; WGCNA modules: CDC; 0 significant genes modules; CD: yellow module with 129 hub genes (*CLCN6*); GO: pathways involved in processes in the vacuole and lysosome
29342213 [48]	11 IA, 11 ctrl	blood neutrophils	mRNA	RNAseq/qPCR	gene expression profiling in peripheral blood neutrophils in IA	DEGs, TermFinder for functional annotation (GO), IPA networks	82 DEGs (up: *MAOA, C21orf15, CYP1B1, ARMC12, CD177*; down: *PRSS21, ETV7, SEPT4, EGR2, GBP1P1*); GO: up: defense response, leukocyte activation, stem cell maintenance, maintenance of cell, stem cell development; down: immune response, immune system process; 4 IPA networks with 7 hub genes (*ERK1/2, AP1, CXCL8, AKT, VEGF*)
30593281 [49]	15 UA, 15 ctrl; testing: 5 UA, 5 ctrl	blood neutrophils	mRNA	RNAseq/qPCR	gene expression profiling in peripheral blood neutrophils in UA, prediction of UA presence	DEGs, GORILLA for functional annotation (GO); classification algorithms	95 DEGs; GO: up: Regulation of defense response, Regulation of inflammatory response, cGMP-mediated signaling, Regulation of response to external stimulus, Negative regulation of defense response; down: Glutathione binding, Tetrapyrrole binding; classification model with 26 transcripts as a potential biomarker for UA
31046777 [41]	19 acute RA, 20 chronic RA, 20 ctrl	peripheral blood cells	mRNA	RNAseq/qPCR	gene expression profiling in RA: acute and chronic	DEGs, Enrichr for functional annotation (GO, WikiPathways, cell-type enrichment), TFBSs (ChIP Enrichment Analysis), mononuclear leukocytes subtypes (flow cytometry)	491 DEmRNAs, **acute RA vs. ctrl**: 403 DEmRNAs: 177 up, 226 down; **chronic RA vs. ctrl**: 0 DEmRNAs; **acute RA vs. chronic RA**: 268 DEmRNAs: 178 up, 290 down: WikiPathways: up: IL-1 Signaling Pathway, Structural Pathway of Interleukin 1 (IL-1), Regulation of toll-like receptor signaling pathway, IL-4 Signaling Pathway, IL-1 signaling pathway, down: G-protein signaling pathways, purine metabolism, inflammatory response pathway, inflammatory response pathway, MAPK signaling pathway; GO-BP: up: MyD88-dependent toll-like receptor signaling pathway, toll-like receptor signaling pathway, pattern recognition receptor signaling pathway, innate immune response-activating signal transduction, activation of innate immune response, down: regulation of lymphocyte activation, regulation of leukocyte activation, T cell differentiation, positive regulation of leukocyte activation, positive regulation of lymphocyte activation; cell type-specific: up: CD33+_Myeloid, CD14+_Monocytes, down: CD4+_Tcells, CD8+_Tcells, CD56+_NKCells, FetalThyroid 721_B_lymphoblasts; TFBSs: up: *SMRT, Nerf2, LXR, FOXM1, AHR*, down: *STAT6, RUNX, MYB, GATA3, MAF*; alternative expression—148 specific gene isoforms (*HEATR1, ACBD6, CCND2, PLEKHA1, ELF2*)
31595394 [44]	29 RA VSP+, 21 RA VSP−	peripheral blood cells	mRNA	Affymetrix microarray	gene expression profiling in peripheral blood cells in RA with/without VSP	DEGs, differential exon expression, alternative splicing, IPA pathways/function	259 DEGs (*ZMAT4, OR2D3, MGC39372, RGS18, ALDH3B2*); 1210 differential exons from 1093 genes (*LMO1, GLDN, HOXB6, ESPL1, DNAH10*); 4 transcripts with alternative splicing (*IL23A, RSU1, PAQR6, TRIP6*); IPA pathways: Cardiac β-adrenergic signaling, α-Adrenergic signaling, Synaptic long-term depression, Synaptic long-term potentiation, GNRH signaling
33059716 [50]	training: 39 UA, 55 ctrl; testing: 16 UA, 24 ctrl	peripheral blood neutrophils	mRNA	RNAseq/qPCR	gene expression profiling in peripheral blood neutrophils in UA, prediction of UA presence	DEGs, IPA networks, GORILLA for functional annotation (GO); classification algorithms	65 DEGs: 42 up, 23 down; GO: up: forebrain anterior/posterior pattern specification, telencephalon cell migration, forebrain cell migration, T cell migration, disruption of cells of other organism; down: regulation of presynaptic membrane potential, motor learning, membrane depolarization during atrial cardiac muscle cell action potential, regulation of systemic arterial blood pressure by aortic arch baroreceptor feedback; IPA networks: cell-to-cell signaling and interaction, nervous system development and function, cell morphology; dermatological diseases and conditions, organismal injury and abnormalities, connective tissue development and function; cell death and survival, connective tissue disorders, inflammatory disease; 37 IA-specific genes (*AC011380.1, C1QL1, CCDC42B, CEP295NL, CERS4*)
33156839 [34]	training: 24 UA, 23 ctrl; testing: 10 UA, 10 ctrl	whole blood	mRNA	RNAseq	gene expression profiling in whole blood in UA, prediction models	DEGs, CIBERSORT for cell composition analysis, GORILLA for functional annotation (GO), IPA networks and pathways; prediction model	18 genes with the greatest predictive value (*ATF3, CBWD6, CCDC85B, CCR8, CHMP4B*); 2 IPA networks: cardiovascular system development and function and tissue development; cancer endocrine system disorders and gastrointestinal disease; CIBERSORT: no statistically significant difference in proportions of cell types; GO for predictive genes: negative regulation of secretion, negative regulation of protein secretion, negative regulation of peptide secretion, cytokine-mediated signaling pathway
34203780 [47]	24 IA, 28 ctrl; validation: 25% of discovery	PBMC	mRNA	RNAseq	expression profiling in PBMC in IA	DEGs, CIBERSORT for cell composition analysis, GOSt for functional annotation (GO), IPA networks, IA risk correlation	54 DEGs: 40 up (*ANKRD24, HLA-DQB2, OR2AK2, PHOSPHO1, ANKRD2*), 14 down (*PHGDH, PDZK1IP1, BOK, RETN, DEFA4*); GO-BP up: biological process, cellular process, biological regulation, regulation of biological process, multicellular organismal process, regulation of cellular process; GO-CC up: cellular component, cellular anatomical entity, cell periphery, plasma membrane, intrinsic component of membrane; GO-MF up: molecular function, binding, protein binding, protein domain specific binding, molecular transducer activity; GO-BP down: multicellular organismal process, cellular process, biological process, response to stimulus; GO-CC down: cellular anatomical entity, cellular component, extracellular region, vesicle, organelle; GO-MF down: binding, molecular function, protein binding, signaling receptor binding; IPA networks: behavior, cell death and survival, connective tissue disorders; amino acid metabolism, cell cycle, cellular development; cardiovascular system development and function, cellular assembly and organization, cellular development; CIBERTSORT: no statistically significant differences in proportions of cell types; risk analysis: *MKRN3* most significantly positively correlated with IA size; *PHGDH* and *TIMD4* most significantly negatively correlated with 5-year rupture risk %
34441376 [37]	31 IA: 37 IA lumen, 31 IA proximal vessels	whole blood	mRNA	qPCR—genes selected based on PMID: 33156839	gene expression in IA lumen vs. proximal parent vessel	DEGs, correlation with IA characteristics	18 DEGs: 6 up (*CBWD6, MT2A, MZT2B, PIM3, SLC37A3*), 3 down (*ST6GALNAC1, TCN2, UFSP1*)
24279374 [51]	6 IA bleb+, 6 IA bleb−, 6 RA, 6 ctrl	plasma	circulating miRNA	Agilent microarray	miRNA expression profiling circulating in plasma in RA, UA w/wo daughter aneurysm	DEmiRNAs, TargetScan for gene prediction, WebGestalt for functional annotation of predicted targets (GO)	**IA bleb+ vs. ctrl**: 68 DEmiRNAs up, 0 down; **IA bleb− vs. ctrl**: 13 DEmiRNAs: 4 up, 9 down; RA vs. ctrl: 15 DEmiRNAs: 2 up, 13 down; common: **UA bleb+ and bleb−) vs. ctrl**: 3 up (miRNA-21, miRNA-22, miRNA-3665); **IA bleb+ and RA vs. ctrl**: 1 up (miRNA-3679-5p); **IA bleb− and RA vs. ctrl**: 5 down (hsa-miR-1471, hsa-miR-3945, hsa-miR-4253, hsa-miR-4314, hsa-miR-574-5p); GO: negative regulation of smooth-muscle cell proliferation, negative regulation of transcription factor activity, vascular endothelial growth factor receptor signaling pathway, actin cytoskeleton organization and biogenesis, negative regulation of transcription from RNA polymerase II promoter
25249297 [52]	20 RA, 20 UA, 20 HC; validation: 93 IA, 50 HC	plasma	circulating miRNA	Agilent miRNA/qPCR	miRNA expression profiling circulating in plasma in IA	DEmiRNAs	99 DEmiRNAs: 69 up (has-let-7d-3p, has-let-7d-5p, hsa-let-7f-5p, hsa-miR-1181, hsa-miR-1227-5p), 30 down (hsa-miR-4644, hsa-miR-4649-3p, hsa-miR-4665-3p, hsa-miR-5100, hsa-miR-6069)
29884860 [33]	14 RA VSP+, 13 RA VSP−, 6 ctrl	peripheral blood	miRNA	RNAseq	miRNA expression profiling in peripheral blood in aSAH with/without VSP	DEmiRNAs, miRTarBase, DIANA, miRTargetLink for target prediction, mirDeep2 for novel miRNA; functional annotation for targets (KEGG)	**RA vs. ctrl**: 8 DEmiRNAs: 3 up (hsa-miR-146a-5p, hsa-miR-589-5p, and hsa-miR-941), 5 down (let-7f-5p, hsa-miR-486-5p, hsa-miR-126-5p, hsa-miR-17-5p, hsa-miR-451a); **RA VSP+ vs. VSP−**: 0 DEmiRNAs; KEGG: Pathways in cancer, PI3K-Akt signaling pathway, HTLV-I infection, Focal adhesion, Proteoglycans in cancer; 33 potential novel miRNAs
31654316 [42]	19 acute RA, 20 chronic RA, 20 ctrl	peripheral blood cells	miRNA	RNAseq	miRNAand target genes expression profiling in RA: acute and chronic	DEmRNAs, DEmiRNAs, miAAE for functional annotation (miRWalk, GO, HMDD2), DEmRNAs, miRBase for target prediction	DEmiRNAs: **acute RA vs. chronic RA vs. ctrl**: 106 mature miRNAs, 90 miRNA precursors; acute RA: up 42 miRNAs, down 39 miRNAs, chronic RA: down 11 miRNAs; miRWalk: Cytokine–cytokine receptor interaction, Translation Factors, Adipogenesis, Parkinson disease, Ubiquitin-mediated proteolysis; HMDD2: Carcinoma Hepatocellular, Carcinoma Non-Small-Cell Lung, Hepatoblastoma, Muscular Disorders Atrophic, Polycythemia Vera; GO: receptor binding, extracellular space, perinuclear region of cytoplasm, protein homodimerization activity, regulation of transcription DNA dependent; 23 predicted targets related to cytokine activity and cytokine–cytokine receptor interactions (*CXCL5, CSF1, FASLG, HMGB1, INHBB*)
31597886 [57]	discovery: 8 RA, 4 UA, 4 HC; validation: 39 RA, 30 UA, 30 HC	plasma	exosomal miRNA	RNAseq/qPCR	expression profiling of exosomal miRNA in IA development and progression	DEmiRNAs	181 DEmiRNAs: **UA vs. ctrl**: 9 up (hsa-miR1296-5p, hsa-miR215-5p, hsa-miR129-5p, hsa-miR200b-3p, hsa-miR3074-5p), 20 down (hsa-miR96-5p, hsa-miR598-3p, hsa-miR202-3p, hsa-miR660-5p, hsa-miR92a-1-5p); **RA vs. ctrl**: 21 up (hsa-let-7a2-3p, hsa-miR1245a, hsa-miR208b-3p, hsa-miR4454, hsamiR-1976), 10 down (hsa-miR874-5p, hsa-miR6874-3p, hsa-miR3146, hsa-miR3529-5p, hsa-miR369-5p); **RA vs. UA**: 92 up (hsa-miR145-5p, hsa-miR202-5p, hsa-miR598-3p, hsa-miR451a, hsa-miR96-5p), 29 down (hsa-miR215-5p, hsamiR-5683, hsa-miR3679-3p, hsa-miR483-5p, hsa-miR6874-3p)
32323261 [54]	discovery: 20 RA, 20 ctrl; validation: 68 RA, 90 ctrl, 20 SAH IA-	plasma	miRNA	Exiqon platform/qPCR	plasma miRNA expression profiling in aSAH	DEmiRNAs, miRWalk for target prediction, DIANA-miRPath for pathways of predicted targets (KEGG), Bingo for functional annotation (GO)	76 DEmiRNAs: 35 up (hsa-miR-122-5p, hsa-miR-192-5p, hsa-miR-215-5p, hsa-miR-99a-5p, hsa-miR-885-5p), 41 down (hsa-miR-328-3p, hsa-miR-28-3p, hsa-miR-18b-5p, hsa-miR-376c-3p, hsa-miR-142-5p); KEGG for 8 candidate miRNAs: Fatty acid biosynthesis, TGF-β signaling pathway, Pathways in cancer, p53 signaling pathway, PI3K-Akt signaling pathway; GO-BP: microtubule organizing center, catabolic process, protein transport, cellular homeostasis, mitochondrion organization; GO-MF: hydrolase activity, lipid binding, carbohydrate binding, receptor activity, phosphoprotein phosphatase activity; GO-CC: cytoskeleton, intracellular, nucleus, cytoskeleton organization, cytoplasmic membrane-bounded vesicle
32922944 [56]	4 RA, 4 UA high risk, 4 UA low risk, 4 ctrl; validation: 10 RA, 10 UA high risk, 10 UA low risk, 10 ctrl	serum	miRNA	Agilent microarray/qPCR	serum miRNA expression profiling in IA, role of miRNA-21	DEmiRNAs, GO for predicted targets of miR-21	77 DEmiRNAs: **RA vs. ctrl**: up: hsa-miR-425, hsa-miR-148b, hsa-miR-27a, hsa-miR-101, hsa-miR-151-5p; down: hsa-miR-3198, hsa-miR-4314, hsa-miR-140-3p, hsa-miR-550a, hsa-miR-148a; miR-21 as potential biomarker of IA formation and rupture
35242102 [55]	65 RA, 55 HC	plasma	miRNA	Agilent microarray/qPCR	miRNA expression profiling in aSAH plasma	DEmiRNAs, TargetScan, PITA, microRNAorg for target prediction, functional annotation (GO, KEGG) of predicted genes	14 DEmiRNAs: microarray: 6 up, 8 down on microarray; validated: 5 down (hsa-miR-23-3p, miR-590-5p, miR-20-5p, miR-142-3p, miR-29b-3p); GO: connective tissue development, angiogenesis, DNA-templated transcription initiation, collagen-activated signaling pathway, muscle tissue development; KEGG: TGF-β-signaling pathway, Hippo signaling pathway, p53 signaling pathway, cellular senescence, AMP-activated protein kinase (AMPK) signaling pathway
31710082 [53]	lncRNAs: 5 IA, 5 HC; validation + mRNA: 30 IA, 30 HC	plasma	lncRNA, mRNA	Arraystar microarray/qPCR	lncRNA expression profiling in plasma in IA, mRNA for CNC networks and functional analyses	DElncRNAs, DEGs, CNC network, functional annotation (GO, KEGG)	797 DElncRNAs: 519 up (TCONS_00000200, ENST00000511927), 278 down (ENST00000421997, ENST00000538202); GO: Negative regulation of striated muscle tissue development, TRNA metabolic process, Transcytosis, Keratinocyte proliferation, Negative regulation of muscle organ development; KEGG: Mineral absorption, Folate biosynthesis, AGE-RAGE signaling pathway in diabetic complications, Platinum drug resistance, Cell adhesion molecules (CAMs); TCONS_00000200 as potential IA biomarker
33023605 [25]	4 IA, 4 STA; for peripheral blood leukocytes 2 tiers: 130 IA, 130 HC	tissue; peripheral blood leukocytes	mRNA, lncRNA	RNAseq/qPCR	mRNA and lncRNA expression profiling in IA plus validation of selected lncRNA expression in peripheral blood leukocytes	DEmRNAs, DElncRNAs, DAVID for functional annotation (GO, KEGG), Cytoscape for CNC network	5 DElncRNAs in peripheral blood leukocytes: lncRNA ENST00000471220, lncRNA ENST00000607042, lncRNA ENST00000478738, MALAT1, lncRNA ENST000000576153; details on tissue analyses presented in Table 1
32939739 [35]	34 IA, 33 ctrl	whole blood	lncRNA	RNAseq	lncRNA expression profiling in whole blood in IA, co-expression analysis	DElncRNAs, IPA networks and pathways, lncRNA ontology database, co-expression networks	263 DElncRNAs; GO-BP: macromolecule metabolism, cellular macromolecule metabolism, RNA processing, regulation of primary metabolism, ncRNA metabolism; GO-CC: DNA package complex, nuclear inner membrane, proteasome complex, spliceosomal complex, small ribosomal subunit; GO-MF: damaged DNA binding, RNA binding, tRNA binding, mRNA binding, nucleoside phosphatase binding; 8 signature lncRNAs for IA (CTC-360G5.6, RP11-72304.9, CTD-2095E4.5, CTA-331P3.1, LINC01226)
33574968 [36]	5 RA, 5 UA, 5 HC	peripheral blood	circRNA	Arraystar microarray/qPCR	circular RNA expression profiling in blood in IA	DEcircRNAs, homemade software for miRNA target prediction, functional annotation (GO, KEGG), Cytoscape for circRNA–miRNA networks, pathways	IA vs. ctrl: DEcircRNAs: 150 up, 85 down; GO-BP: positive regulation of cellular process, homophilic cell adhesion via plasma membrane adhesion molecules, cell–cell signaling, cell–cell adhesion via plasma-membrane adhesion molecules, positive regulation of cellular metabolic process; KEGG: human papillomavirus infection, proteoglycans in cancer, pathways in cancer, hepatocellular carcinoma, autophagy—animal
33603847 [46]	3 multiple IA, 3 HC	PBMC	circRNA	RNAseq/qPCR	circRNA expression profiling in PBMC in multiple IA	DEcircRNAs, functional annotation (GO, KEGG), TargetScan, circBank, miRanda, miRTarBase for circRNA–miRNA–mRNA network construction, ceRNA network	60 DEcircRNAs: 20 up (hsa_circ_0135895, hsa_circ_0008911, hsa_circ_0008122, hsa_circ_0074837, hsa_circ_0078380), 40 down (hsa_circ_0009076, hsa_circ_0000982, hsa_circ_0001492, hsa_circ_0000698, hsa_circ_0141172); GO-BP up: negative regulation of execution phase of apoptosis, extracellular negative regulation of signal transduction, negative regulation of signaling receptor activity, drug metabolic process, hydrogen peroxide catabolic process; GO-CC up: BLOC-1 complex, intracellular, mitochondrial matrix, mitochondrion, cytoplasm; GO-MF up: receptor antagonist activity, receptor inhibitor activity, thiaoredaoxin peroxidase activity, peroxiredoxin activity, oxidoreductase activity; KEGG up: thiamine metabolism, antigen processing and presentation, protein digestion and absorption, Fanconi anemia pathway, amoebiasis; GO-BP down: metabolic process, cellular metabolic process, cellular nitrogen compound metabolic process, macromolecule metabolic process, primary metabolic process; GO-CC down: intracellular, intracellular organelle, intracellular membrane-bounded organelle, membrane-bounded organelle, cytoplasm; GO-MF down: protein binding, peptide binding, amide binding, enzyme binding, RNA binding; KEGG down: leukocyte transendothelial migration, viral carcinogenesis, protein processing in endoplasmic reticulum, natural killer cell-mediated cytotoxicity; ceRNA networks: 3 circRNAs (predicted miRNAs): hsa_circ_0135895 (hsa-miR-619-3p, hsa-miR-4324, hsa-miR-5687, hsa-miR-3529-5p, hsa-miR-379-5p), hsa_circ_0000682 (hsa-miR-448, hsa-miR-1248, hsa-miR-302a-5p, hsa-miR-627-3p, hsa-miR-1248), hsa_circ_0000690 (hsa-miR-4726-3p, hsa-miR-4520-3p, hsa-miR-4514, hsa-miR-4692, hsa-miR-6842-3p) and regulated genes: *PTK2, PRKCB, ITGAL*
32424559 [43]	19 acute RA, 20 chronic RA, 20 ctrl	peripheral blood cells	sRNA	RNAseq	small RNA expression profiling in RA acute and chronic	DEsRNAs (piRNA, tRNA, scRNA, snoRNA, rRNA, miRNA), conservation analysis (phastCons), TFBSs (seqinspector)	542 DEsRNAs (108 piRNAs, 99 rRNAs, 90 miRNAs, 43 scRNAs, 36 tRNAs, 32 snoRNAs), 105 DEsRNAs in RA acute, 77 DEsRNAs in RA chronic, 286 DEsRNAs in RA vs. ctrl; RA: up: miRNAs, down: piRNAs, rRNAs; TFBSs: *GR, RXRA, ERALPHA*

aSAH, aneurysmal subarachnoid hemorrhage; BP, biological process; CC, cellular component; ctrl, control; DCI, delayed cerebral ischemia; DEcircRNAs, differentially expressed circRNA; DEGs, differentially expressed genes; DElncRNAs, differentially expressed lncRNA; DEmiRNAs, differentially expressed miRNAs; DEmRNAs, differentially expressed mRNAs; ECM, extracellular matrix; GO, Gene Ontology; HC, healthy control; IA, intracranial aneurysm; IPA, Ingenuity Pathway Analysis; KEGG, Kyoto Encyclopedia of Genes and Genomes; MF, molecular function; PBMC, peripheral blood mononuclear cell; qPCR; quantitative PCR; RA, ruptured aneurysm; RNAseq, RNA sequencing; STA, superficial temporal artery; UA, unruptured aneurysm; VSP, vasospasm; WGCNA, Weighted Gene Co-Expression Network Analysis. In some studies, GO and KEGG terms were not analyzed separately for up- and down-regulated DERNAs but only for DERNAs as a whole. Data presented in the table reflect available data.

**Table 3:** Studies on RNA expression in the intracranial aneurysm wall utilizing existing datasets.

PMID/Reference	Datasets ID	Cohorts	RNA Type	Detection/Verification Methods	Aim of the Study	Analytical Methods	Major Findings including Differentially Expressed RNAs, Involved Pathways/Functions (Top 5)
23740452 [58]	GSE13353	11 RA, 8 UA	mRNA	Affymetrix microarray	gene expression profiling in RA and UA	DEGs, DAVID, GSEA for functional annotation (GO, KEGG), STRING for PPI network, TfactS for TF prediction, Sylamer for associated miRNAs prediction	2119 DEGs: 1062 up in RA, 1057 down in RA; GO: inflammatory response, response to wounding, defense response; PPI: GRB2, PPP2R2B; TFs: NFKB1, HIF1A, SP1, JUN; predicted miRNAs: miR-33a-5p, miR-659-3p, miR-524-5p, miR-661, miR-1207-5p
24065667 [59]	GSE26969	3 UA, 3 STA	mRNA	Affymetrix microarray	gene expression profiling in UA	DEGs, functional annotation with PATHWAY (KEGG), BiNGO (GO), TRANSFAC, TRED for regulation network (TFs)	3661 DEGs; GO: antigen processing and presentation of peptide or polysaccharide antigen via MHC II class, response to organic substance, antigen processing and presentation, multicellular organismal homeostasis, negative regulation of RNA metabolic processes, negative regulation of macromolecule metabolic process; KEGG: adherens junction, phosphatydylinositol signaling system, ribosome, cicardian rhythm, Parkinson’s disease; 7 TFs (*STAT1, FLT1, ETS2, SMAD2, ADD1*) with 15 DEGs—16 regulatory relationships
24615040 [60]	GSE26969	3 UA, 3 STA	mRNA	Affymetrix microarray	gene expression profiling in UA	DEGs, STRING for PPI network, FuncAssociate for functional annotation (GO)	169 DEGs: 4 up, 165 down; GO: Muscle contraction, Muscle system process, Regulation of muscle contraction, Regulation of muscle system process, Actomyosin structure organization; PPI network with MYH11 as a main hub gene
25721208 [67]	GSE54083 GSE15629	16 RA, 11 UA, 15 ctrl (STA, MMA)	mRNA	Affymetrix, Agilent microarray	gene expression profiling, interaction networks in IA	DEGs, functional annotation (GO, KEGG), STRING for PPI network, WGCNA for functional modules	**RA vs. ctrl**: 452 DEGs: 299 up, 153 down; GO up: Cartilage condensation, Response to transforming growth factor-β, Cellular response to transforming growth factor-β stimulus, Response to calcium ion, Response to mineralocorticoid, GO down: Cellular response to temperature stimulus, Response to prostaglandin D, Cellular response to prostaglandin D stimulus, Intestine smooth-muscle contraction, Gastrointestinal system smooth-muscle contraction; KEGG up: Osteoclast differentiation, Arginine and proline metabolism, RNA transport, Chagas disease (American trypanosomiasis); PPI networks with 238 nodes (hub genes: *FOS, GCG, NTS, CASR*); WGCNA: grey module (GO: response to wounding, extracellular structure organization, immune response, cell adhesion, biological adhesion; KEGG: ECM–receptor interaction, arrhythmogenic right ventricular cardiomyopathy (ARVC)); **UA vs. ctrl**: 570 DEGs: 312 up, 258 down; GO up: Regulation of vasculature development, Carbohydrate-mediated signaling, Osteoclast differentiation, Organ formation, Immune system process; GO down: Negative regulation of calcium ion transmembrane transporter activity, Apolipoprotein A-I-mediated signaling pathway, Regulation of release of sequestered calcium ion into cytosol by sarcoplasmic reticulum, Regulation of ryanodine-sensitive calcium-release channel activity, Regulation of cardiac muscle cell membrane potential; KEGG up: Cytokine–cytokine receptor interaction, Arginine and proline metabolism, Rheumatoid arthritis, Glycosaminoglycan biosynthesis-keratan sulfate, ECM–receptor interaction KEGG down: Spliceosome, Protein processing in endoplasmic reticulum, Mucin-type O-glycan biosynthesis; PPI networks with 161 nodes (hub genes: *FOS, NTS, CD68, GCG, ALPP*); WGCNA: yellow module (GO: male sex determination, response to vitamin D, sex determination, response to temperature stimulus, membrane invagination; KEGG: cytokine–cytokine receptor interaction)
29115560 [61]	GSE54083	8 RA, 10 STA	mRNA	Agilent microarray	gene expression profiling in IA, regulation with miRNA, TFs	DEGs, DAVID for functional annotation (GO, KEGG), STRING, CytoNCA for PPI network, miRNA target genes prediction (MiRwalk2, MiRDB, RNA22, miRanda, RNAhybrid, TargetScan), TF prediction (ITFP, TRANSFAC), TF-target-miRNA network (Cytoscape)	777 DEGs: 402 up, 275 down; GO up: cellular respiration, regulation of programmed cell death, respiratory electron transport chain, energy derivation by oxidation of organic compounds, immune system development; GO down: neuron projection morphogenesis, cellular component morphogenesis, neuron projection development, cell morphogenesis involved in differentiation, cell morphogenesis; KEGG up: primary immunodeficiency, asthma, Huntington disease, Alzheimer disease, cellular response; KEGG down: pathways in cancer, melanogenesis, natural killer cell-mediated cytotoxicity; PPI network (nodes: CD40, CD40LG, DRD2, TGFB1); DEGs as TFs: *ARHGAP25, CCNE1, CIAO1, CIRBP, STF*; 12 IA associated miRNAs (hsa-miR-125a, hsa-miR-125b, hsa-miR-145, hsa-miR-146a, hsa-miR-21)
29328431 [62]	GSE26969	3 UA, 3 STA	mRNA	Affymetrix microarray	gene expression profiling in UA	DEGs, DAVID for functional annotation (GO, KEGG), BIND, ClusterOne for PPI network, TFs regulatory network	1124 DEGs: 989 up (*MMP16, SOX4, NUFIP2, TWIST1, COL5A2*), 135 down (*PLN, ADH1C, MYL9, SORBS1*); GO: RNA binding, organelle lumen, membrane-enclosed lumen, nuclear lumen, RNA splicing, mRNA metabolic process; KEGG: splicesosome; PPI network (HFN4A, ORC2L, MAFK, JUN); 6 TFs (*HNF6, HNF4A, E2F4, YY1, H4*) and 24 DEGs in TFS regulatory network; regulatory pathway *HFN6-HFN4-E2F4*
29552131 [69]	GSE13353 GSE15629	19 RA, 14 UA	mRNA	Affymetrix microarray	gene expression profiling in RA and UA, genes critical for rupture	DEGs, functional annotation (KEGG), PPI network (Biological General Repository for Interaction Datasets, Human Protein Reference Database, Database of Interacting Proteins)	1,029 DEGs: 527 up, 502 down; KEGG: MAPK signaling pathway, Pathways in cancer, NOD-like receptor signaling pathway, ErbB signaling pathway, Cysteine and methionine metabolism; PPI network with 510 nodes (FN1, A4, APP, NXF1, STAT3)
30366668 [70]	GSE15629 GSE54083 GSE13353 GSE6551 GSE26969 GSE36791	vessel wall: 31 RA, 23 UA; blood: 43 RA, 18 UA	mRNA	Affymetrix, Agilent, Illumina microarray	gene expression profiling in RA	DEGs, clusterProfiler for functional annotation (GO, KEGG), STRING for PPI network, MCODE for subnetworks; common DEGs for tissue and blood samples	158 DEGs; GO: Antigen processing and presentation of peptide or polysaccharide antigen via MHC class II, Cellular response to interferon-γ, Interferon-γ-mediated signaling pathway, Antigen processing and presentation of exogenous peptide antigen via MHC class II, MHC class II protein complex assembly 3; KEGG: Th17 cell differentiation, Th1 and Th2 cell differentiation, systemic lupus erythematosus, Staphylococcus aureus infection, rheumatoid arthritis; PPI network with 155 nodes; 9 common key genes for tissue and blood (*BASP1,CD74, CEBPB, ECHDC2, GZMK*)
31329646 [71]	GSE13353 GSE15629	19 RA, 14 UA	mRNA	Affymetrix microarray	gene expression profiling in RA, drug candidates for rupture prevention	co-expression networks with WGCNA modules, DAVID for functional annotation (GO), computational drug repurposing (L1000), PSEA (population specific expression analysis), GoSemSim	12 WGCNA modules (4 mapped to immune function); 164 module-based drug/compound candidates (prostratin, tereic-acid, phorbol-12-myristate-13-acetate, ingenol, MLN-4924); GO for cell types (PSEA): UA: macrophage (immune response, inflammatory response, innate immune response, cellular response to lipopolysaccharide, leukocyte migration), T cell (T cell activation, cell surface receptor signaling pathway, T cell differentiation, regulation of immune response, adaptive immune response), smooth-muscle (platelet aggregation, muscle organ development, muscle contraction); RA: macrophage (immune response, peptide/polysaccharide presentation via MHC II, exogenous peptide presentation via MHC II, inflammatory response, antigen processing and presentation), T cell (T cell activation, regulation of immune response, cell surface receptor signaling pathway, immune response, T cell receptor signaling pathway)
31238169 [72]	GSE75436 GSE6551 GSE26969 GSE13353	42 IA, 18 ctrl	mRNA	Affymetrix microarray	gene expression profiling in IA, IA formation	DEGs, clusterProfiler for functional annotation (GO, KEGG), GSEA, STRING for PPI network, MCODE for subnetworks	114 DEGs: 43 up, 71 down; GO-BP: muscle system process, muscle contraction, muscle cell differentiation, regulation of muscle contraction, regulation of muscle system process; GO-CC: proteinaceous extracellular matrix, contractile fiber part, contractile fiber, sarcomere, myofibril; GO-MF: cytokine activity, G-protein-coupled receptor binding, structural constituent of muscle, structural constituent of cytoskeleton, insulin-like growth factor binding; KEGG: calcium signaling pathway, neuractive ligand-receptor interaction, complement and coagulation cascades, vascular smooth-muscle contraction, apelin signaling pathway; GSEA: vascular smooth-muscle cell proliferation, smooth-muscle contraction, complement activation, complement receptor-mediated signaling pathway, vascular smooth-muscle contraction; PPI network with 50 nodes (MYH11, CNN1, MYOCD, ACTA1, LMOD1)
31545495 [63]	GSE75436	15 IA, 14 STA	mRNA	Affymetrix microarray	gene expression profiling in IA	DEGs, DAVID, GSEA for functional annotation (GO, KEGG), STRING for PPI network	782 DEGs: 392 up, 390 down; GO-BP up: inflammatory response, immune response, cell adhesion, extracellular matrix organization, neutrophil chemotaxis; GO-BP down: muscle contraction, nervous system development, cell adhesion, smooth-muscle contraction, neurotransmitter catabolic process; GO-CC up: plasma membrane, extracellular region, extracellular space, integral component of plasma membrane, collagen trimer; GO-CC down: Z disc, proteinaceous extracellular matrix, actin cytoskeleton, sarcolemma, sarcoplasmic reticulum membrane; GO-MF up: extracellular matrix structural constituent, IgG binding, chemokine activity, coreceptor activity, carbohydrate binding; GO-MF down: actin binding, structural constituent of muscle, ion channel binding, cytoskeletal protein binding, primary amine oxidase activity; KEGG: staphylococcus aureus infection, amoebiasis, phagosome, leishmaniasis, ECM–receptor interaction; PPI network with 33 nodes (TNF, IL8, TLR4, PLCB4, AGTR1)
33222929 [73]	GSE75436 GSE54083	28 IA, 20 ctrl STA	mRNA	Affymetrix, Agilent microarray	gene expression profiling in IA	DEGs, DAVID for functional annotation (GO, KEGG), STRING, CytoNCA for PPI networks and module, MCODE for subnetworks, Enrichr tool for miRNAs-DEGs, TRANSFAC, ITFP for TFs prediction, TF-miRNA-target regulatory network construction	1332 DEGs: 720 up, 612 down; GO up: chemotaxis, inflammatory response, response to wounding, defense response, immune response; GO down: regulation of blood pressure, regulation of ehart rate, cell adhesion, muscle filament sliding, muscle contraction; KEGG up: allograft rejection, cytokine–cytokine interaction, ECM–receptor interaction, intestinal immune network for IgA production, cell adhesion molecules (CAMs); KEGG down: calcium signaling pathway, adrenergic signaling in cardiomyocytes, focal adhesion, cGMP-PKG signaling pathway, vascular smooth-muscle contraction; PPI networks: up: 539 nodes (TNF, PTPRC, IL8, IL10, TYROBP); down: 385 nodes (CALM1, ACTA1, ACTN2, ACTA2, ACTC1); miRNA: 7 for up DEGs, 14 for down DEGs; 17 TFs for up DEGs; 22 TFs for down DEGs; *VCAM1, TNF, CTSS, IL10, IL1B, IL6*, miR-19A/B/C as potential IA biomarkers
33313152 [74]	GSE13353 GSE15629 GSE54083 GSE122897	24 RA, 18 UA	mRNA	Affymetrix, Agilent, Illumina microarray	gene expression profiling in RA	DEGs, co-expression networks with WGCNA modules, DAVID for functional annotation (GO, KEGG), MCODE for key gene cluster, GSEA for key genes	49 DEGs: 28 up (*PPBP, PF4, S100A8, FPR1, C15orf48*); 21 down (*NR1D2, ATP1A2, FMO2, SLC6A1, CYP4X1*); **RA**: GO-BP: Signal transduction, Inflammatory response, Immune response, Innate immune response, Defense response to bacterium; KEGG: Cytokine–cytokine receptor interaction, Chemokine signaling pathway; WGCNA modules (hub gene): 8 gene modules for RA: Blue (*SLC7A7*), Cyan (*GFPT2*), Green (*ARF4*), Green yellow (*MAL*), Lightcyan (*KLHL34*), Midnightblue (*PLCB4*), Purple (*PRKG1*), Salmon (*VNN2*); 6 gene modules for **UA**: Brown (*BCHE*), Lightcyan (*LNPEP*), Lightgreen (*MT1G*), Purple (*MYADM*), Salmon (*CD53*); KEGG: RA: Blue Brown: Lysosome, Tuberculosis, Phagosome, Osteoclast differentiation, Chemokine signaling pathway; Cyan: Pathways in cancer, HTLV-I infection, PI3K-Akt signaling pathway, Proteoglycans in cancer, Focal adhesion; Green: Protein processing in endoplasmic reticulum, PI3K-Akt signaling pathway, Focal adhesion, ECM–receptor interaction, Proteoglycans in cancer; Green-yellow: Arginine and proline metabolism, Gastric acid secretion, Histidine metabolism, Prion diseases, Mineral absorption; Midnightblue: Pathways in cancer, Amoebiasis, Calcium signaling pathway, Inositol phosphate metabolism, ECM–receptor interaction; Purple: Pathways in cancer, PI3K-Akt signaling pathway, Regulation of actin cytoskeleton, Focal adhesion, cGMP-PKG signaling pathway; Salmon: Cytokine–cytokine receptor interaction, Chemokine signaling pathway; UA: Brown: PI3K-Akt signaling pathway, Cell adhesion molecules (CAMs), Focal adhesion, Retrograde endocannabinoid signaling, Glutamatergic synapse; Lightcyan: Pathways in cancer, PI3K-Akt signaling pathway, Proteoglycans in cancer, Regulation of actin cytoskeleton, Focal adhesion; Lightgreen: Mineral absorption, Glycolysis/Gluconeogenesis, Biosynthesis of antibiotics, Biosynthesis of amino acids, Carbon metabolism; Purple: Focal adhesion, ECM–receptor interaction, PI3K-Akt signaling pathway, Leukocyte transendothelial migration, Regulation of actin cytoskeleton; Salmon: Focal adhesion, ECM–receptor interaction, PI3K-Akt signaling pathway, Leukocyte transendothelial migration, Regulation of actin cytoskeleton; Key genes for RA: *C15orf48, AQP9, SLA, MPP1, PDZRN3*
32589050 [75]	GSE6551 GSE13353 GSE26969 GSE75436 GSE106520 GSE36791	vessel wall: 33 IA, 27 ctrl; serum: 16 UA, 16 ctrl; blood: 43 RA, 18 UA	mRNA	Affymetrix, Agilent, Ilumina microarray	gene expression profiling in IA—vessel wall and blood	DEGs, co-expression networks with WGCNA modules, clusterProfiler for functional annotation (GO, KEGG)	UA vs. ctrl: DEGs: 783 up, 1097 down; WGCNA modules: purple, green-yellow, yellow; GO: purple: signal release, regulation of neuron projection development, regulation of hormone secrection, regulation of amino acid transport, positive regulation of secretion by cells, green-yellow: protein retention in ER lumen, nucleobase-containing small molecule biosynthese process, maintenance of protein localization in organelle, yellow: regulation of plasma lipoprotein particle levels, receptor catabolic process, plasma lipoprotein particle clearence, neutrophil-mediated immunity, neutrophil degranulation; KEGG: purple: steroid hormone biosynthesis, Ras signaling pathway, mannose type O-glycan biosynthesis, glyoxylate and dicarboxylate metabolism, glycosaminoglycan biosynthesis—heparan sulfate, green-yellow: nucleotide excision repair, mismatch repair, glutathione metabolism, fructose and mannose metabolism, DNA replication, yellow: riboflavin metabolism, platinum drug resistance, pentose and glucuronate interconversion, other glycan degradation, N-glycan biosynthesis; RA vs. ctrl: DEGs: 711 up, 1020 down; WGCNA modules: blue, turquoise; GO: blue: translational inhibition, SRP-dependent cotranslational protein targeting to membrane, protein targeting to membrane, protein targeting to ER, protein targeting, turquoise: regulation of protein serine/threonine kinase activity, regulation of innate immune response, reactive oxygen species metabolic process, positive regulation of hemopoiesis, positive regulation of cell activation; KEGG: blue: starch and sucrose metabolism, spliceosome, ribosome, proteosome, proponoate metabolism, turquoise: Toll-like receptor signaling pathway, platelet activation, phagosome, osteoclast differentiation, NOD-like receptor signaling pathway; 24 hub genes expression in blood consistent with tissue; potential circulating markers for RA: *CD163, FCEREG, FPRT1, ITGAM, NLRC4*
34354366 [76]	GSE15629, GSE75436 GSE26969 GSE6551 GSE122897	34 IA, 26 ctrl; validation: 44 IA, 16 ctrl	mRNA	Affymetrix microarray RNA, RNAseq	gene expression profiling in IA, potential hub genes and pathways in IA	DEGs, RRA (Robust Rank Aggregation), clusterProfiler for functional annotation (GO, KEGG), STRING for PPI network, cytoHubba (hub genes)	RRA: 136 DEGs: 45 up (*DSP, KRT14, FAP, COL5A2, ARL4C*), 91 down (*RERGL, NPY1R, PDZRN4, AOC3, RBPMS2*); GO: extracellular matrix structural constituent, extracellular matrix structural constituent conferring tensile strength, glycosaminoglycan binding, carbohydrate binding, structural constituent of muscle; KEGG: ECM–receptor interaction, protein digestion and absorption, phenylalanine metabolism, cAMP signaling pathway, amphetamine addiction; PPI: 8 hub genes associated with IA development: *VCAN, COL1A1, COL11A1, COL5A2, POSTN, THBS2, CDH2*
34403136 [65]	GSE122897	21 RA, 21 UA, 16 cortical artery	mRNA	RNAseq	gene expression profiling in RA and UA	DEGs, g:GOSt tool in the g:Profiler, REVIGO for functional annotation (GO), IPA networks; validation with available data: PRJNA553307, PRJNA665639, PMID: 29729990, PMID: 32525733, PMID: 25528428	total 1768 DEGs, 318 DEGs in multiple comparisons; **UA vs. ctrl**: 377 DEGs: 123 up (*COL10A1, IGHM, PMEL, SELP, PLVAP*), 254 down (*B3GAT1, SLC6A13, SLC13A4, GPR37L1, PTGDS*); GO-BP up: extracellular matrix organization, extracellular structure organization, cell motility, localization of cell, skeletal system development; GO-MF up: extracellular matrix structural constituent, structural molecule activity, glycosaminoglycan binding; GO-CC up: extracellular matrix, extracellular region, endoplasmic reticulum lumen, collagen type IV trimer; GO-BP down: chemical synaptic transmission, regulation of neurotransmitter levels, neurotransmitter transport, cell–cell signaling, nervous system development; GO-MF down: metal ion transmembrane transporter activity, transmembrane transporter activity, inorganic solute uptake transmembrane transporter activity, transporter activity, active ion transmembrane transporter activity; GO-CC down: synapse, cell junction, presynapse, cell periphery, cell projection; IPA networks: Amino acid metabolism, molecular transport, small molecule biochemistry; behavior, cellular function and maintenance, small molecule biochemistry; endocrine system disorders, organ morphology, organismal injury and abnormalities; cell morphology, lipid metabolism, small molecule biochemistry; carbohydrate metabolism, connective tissue development and function, skeletal and muscular system development and function; cell morphology, connective tissue development and function, skeletal and muscular system development and function; **RA vs. ctrl**: 925 DEGs: 349 up (*CXCL5, ACAN, LAMC2, PLAC8, CD300E*), 576 down (*KIF1A, PSD2, RUNDC3A, BCAN, CAMK2A*); GO-BP up: immune system process, response to cytokine, extracellular matrix organization, extracellular structure organization, immune response; GO-MF up: oxidoreductase activity, acting on paired donors with incorporation or reduction of molecular oxygen, 2-oxoglutarate as one donor, and incorporation of one atom each of oxygen into both donors, dioxygenase activity, extracellular matrix structural constituent; GO-CC up: endomembrane system, endoplasmic reticulum lumen, extracellular matrix, cytoplasmic vesicle, specific granule; GO-BP down: nervous system development, chemical synaptic transmission, cell–cell signaling, regulation of neurotransmitter levels, neurotransmitter transport; GO-MF down: metal ion transmembrane transporter activity, inorganic solute uptake transmembrane transporter activity, transmembrane transporter activity, transporter activity, cytoskeletal protein binding; GO-CC down: synapse, cell junction, neuron projection, cell projection, postsynapse; IPA networks: Cellular development, embryonic development, organismal development; cancer, hematological disease, organismal injury and abnormalities; cell-to-cell signaling and interaction, molecular transport, nervous system development and function; cell-to-cell signaling and interaction, cellular assembly and organization, cellular development; cardiovascular disease, organismal injury and abnormalities, tissue morphology; **RA vs. UA**: 466 DEGs: 383 up (*MTRNR2L1, CD300E, MARCO, ANPEP, CLEC5A*), 83 down (*CRLF1, KIF1A, KRT17, HMCN2, THBS4*); GO-BP up: immune response, immune system process, cell activation, leukocyte degranulation, secretion by cell; GO-MF up: cargo receptor activity, Toll-like receptor binding, identical protein binding, Rac GTPase binding, cytokine binding; GO-CC up: plasma membrane, cell periphery, secretory granule, vesicle, membrane; GO-BP down: multicellular organismal process, system development; IPA networks: Cellular development, cellular function and maintenance, cellular growth and proliferation; carbohydrate metabolism, cellular movement, hematological disease; cell-to-cell signaling and interaction, hematological system development and function, hypersensitivity response; carbohydrate metabolism, cell morphology, inflammatory response; cellular development, cellular growth and proliferation, hematological system development and function; in validation—common genes/protein: *ALDH1A1, HMOX1, PPIF, TYMP*
34895131 [77]	Aneurysm Gene Database www.cuilab.cn/agd (accessed on 31 December 2022)	different types of aneurysms: IA, AAA, TAA, TAAD, AA, AD, RA	mRNA	microarray RNA	gene expression profiling and protein–protein interaction networks in different types of aneurysms	PPI networks, DEGs, clusterProfiler for functional annotation (GO, KEGG)	**IA**: GO-MF: ubiquitin protein ligase binding, ubiquitin-like protein ligase binding, phosphatase binding, RNA polymerase II transcription factor binding; GO-BP: regulation of DNA-binding transcription factor activity, regulation of apoptotic signaling pathway, regulation of binding, positive regulation of DNA-binding transcription factor activity, peptidyl-serine phosphorylation; KEGG: hepatitis B, viral carcinogenesis, Kaposi sarcoma-associated herpesvirus infection, Ebstein-Barr virus infection, proteoglycans in cancer; **RA**: GO-MF: ubiquitin ligase binding, ubiquitin-like protein ligase binding, disordered domain specific binding, phosphatase binding, protein phosphatase binding; GO-BP: regulation of binding, regulation of protein binding, response to heat, positive regulation of proteolysis, response to reactive oxygen species; KEGG: fluid shear stress and atherosclerosis, prostate cancer, proteoglycans in cancer, PI3K-Akt signaling pathway, viral carcinogenesis; candidate driver genes: **IA**: *CUL3, JUN, CAV1, WWOX, EGFR*; **RA**: *TXN, HP, MMP9, YWHAQ, GRB2*
34997174 [78]	GSE13353 GSE54083 GSE75436	total: 11 RA, 23 UA, 15 STA	mRNA	Affymetrix, Agilent microarray	epithelial–mesenchymal transition genes expression in UA	DEGs focused on EMT-related genes (900); co-expression network with WGCNA modules, clusterProfiler for functional annotation (GO, KEGG), STRING for PPI, GSEA for hub genes	**RA vs. ctrl**: DEGs: 61 up (*SDC1, HK2, TIMP1, HAVCR2, CCR5*), 15 down (*SERPINI1, ADIPOQ, AGTR1, AFAPIL2, WNT11*); GO-BP: response to lipopolysaccharide, response to molecule of bacterial origin, cellular response to lipopolysaccharide, cellular response to molecule of bacterial origin, cellular response to biotic stimulus; GO-CC: collagen-containing extracellular matrix, secretory granule lumen, cytoplasmic vesicle lumen, vesicle lumen, external side of plasma membrane; GO-MF: receptor-ligand activity, signaling receptor activator activity, cytokine activity, G-protein-coupled receptor binding, cytokine receptor binding; KEGG: chemokine signaling pathway, proteoglycans in cancer, lipid and atherosclerosis, shigellosis, viral protein interaction with cytokine and cytokine receptor; **RA vs. UA**: DEGs: 35 up (*CD36, WNT11, HAS2, PDGFD, MYC*), 8 down (*NUAK1, CDH11, DLX2, FZD7, VCAN*); GO-BP: regulation of vasculature development, epithelial cell proliferation, ameboidal-type cell migration, regulation of angiogenesis, urogenital system development; GO-CC: collagen-containing extracellular matrix, endoplasmic reticulum lumen, transcription regulator complex, focal adhesion, cell-substrate junction; GO-MF: receptor ligand activity, signaling receptor activator activity, cytokine activity, cytokine receptor binding, G-protein-coupled receptor binding; KEGG: proteoglycans in cancer, PI3K-Akt signaling pathway, human cytomegalus virus infection, viral protein interaction with cytokine and cytokine receptor, non-alcoholic fatty liver disease; **UA vs. ctrl**: DEGs: 40 up (*CEMIP, CDKN2A, CDH2, CDH11, SALL1*), 30 down (*ADIPOQ, WNT11, GPC3, CCL21, HAS2*); GO-BP: ossification, urogenital system development, renal system development, cell chemotaxis, epithelial tube morphogenesis; GO-CC: collagen-containing extracellular matrix, endoplasmic reticulum lumen, membrane raft, membrane microdomain, membrane region; GO-MF: receptor ligand activity, signaling receptor activator activity, cytokine activity, G-protein-coupled receptor binding, cytokine receptor binding; KEGG: cytokine–cytokine receptor interaction, proteoglycans in cancer, Salmonella infection, viral protein interaction with cytokine and cytokine receptor, malaria; 3 common gene for all: *ADIPOQ, WNT1, CCL21*; WGCNA modules: green positively correlated with ctrl and negatively with UA; red negatively correlated with ctrl and positively with UA; GSEA hub genes: green module: *WNT11, GLI1, PCDH9, GPC3, L1CAM*; red module: *KRT18, CTHRC1, POSTN, CDH11, FHL2*; PPI hub genes: *CDH11, SPARC, FSTL1, FN1, PCDH9*
35250300 [79]	GSE13353 GSE54083 GSE75436	47 IA, 25 STA	mRNA	Affymetrix, Agilent microarray	gene expression profiling in IA	DEGs, WebGestalt for functional annotation (GO, KEGG), co-expression network with WGCNA modules, STRING for PPI networks, CytoHubba for hub genes, CIBERSORT for infiltrating cell composition analysis	266 DEGs: 162 up (*COL11A1, EME2, ADAMTS10, HTR4, DAPLI*), 104 down (*CASQ2, ITLN1, RBPMS2, MYOT, ACTAI*); GO-BP: inflammatory response, defense response, immune response, muscle system process, muscle contraction; KEGG: chemokine signaling pathway, rheumatoid arthritis, glycine, serine and threonine metabolism, Toll-like receptor signaling pathway; WGCNA modules (pathways): blue (immune response, inflammatory response, leukocyte activation, chemokine signaling pathway, Toll-like receptor signaling pathway), pink (inflammatory response, defense response, myeloid leukocyte cytokine production, cytokine production involved in immune response, regulation of mast cell cytokine production, regulation of inflammatory response, positive regulation of immune system process, leukocyte-mediated immunity, mast cell cytokine production, regulation of intreleukin-10 production, complement and coagulation cascades); Hub genes: blue module: *CCR5, CCL20*, pink module: *FPR3, CCL4*; CIBERSORT: macrophages, neutrophils proportions higher in ctrl, M0, M2 macrophages, activated mast cells proportions higher in IA
35432454 [80]	GSE13353 GSE15629 GSE54083	27 RA, 19 UA	mRNA	Affymetrix, Agilent microarray	gene expression profiling in RA	DEGs, DAVID for functional annotation (GO, KEGG), STRING for PPI network, CytoHubba for hub genes, MCODE for subnetworks	249 common DEGs: 96 up, 153 down; GO-BP up: Positive regulation of cell proliferation, Apoptotic process, Response to lipopolysaccharide, Inflammatory response, Negative regulation of cell proliferation; GO-CC up: Nucleoplasm, Membrane, Endoplasmic reticulum, Endoplasmic reticulum membrane, Cell surface; GO-MF up: Protein binding, Receptor activity, SH3 domain binding, KDEL sequence binding, ER retention sequence binding; KEGG up: Proteoglycans in cancer, Cytokine–cytokine receptor interaction, Mineral absorption, Hepatitis B, Malaria; GO-BP down: Positive regulation of transcription from RNA polymerase II promoter, Protein phosphorylation, Positive regulation of gene expression, Positive regulation of apoptotic process, Axonogenesis; GO-CC down: Extracellular exosome, Receptor complex, Myelin sheath, Endosome membrane, Mitochondrial membrane; GO-MF down: Metal ion binding, ATP binding, Kinase activity, NADP binding, Steroid hormone binding; KEGG down: Focal adhesion, Pancreatic secretion, Thyroid hormone signaling pathway, Staphylococcus aureus infection, ErbB signaling pathway; PPI network: 241 nodes (STAT3, APP, JUN, ITGB2, GSK3B); potential biomarker for RA—hub genes: *APP, JUN, GSK3B, ErbB2, PPBP, THBS1*
35465608 [81]	GSE75436 GSE54083 GSE26969 GSE13353 GSE15629 GSE158558 GSE122897 GSE66240	108 IA, 73 ctrl; training: microarray; validation: RNAseq	mRNA	Affymetrix, Agilent, Illumina microarray, RNAseq	expression of endoplasmic reticulum stress-related genes in IA	DEGs, GSEA for functional annotation (GO, KEGG), ERS (endoplasmic reticulum stress)-related DEGs, and pathways, immunocyte infiltration, VSMC phenotype, co-expression analysis with WGCNA modules, ERS-TF-miRNA networks	DEGSs: training: 1628 up, 2013 down; validation: 590 up, 685 down; ERS-related DEGs: 6 up (*FKBP14, TOR1A, EDEM1, BAX, CALR, SEC61B*), 2 down (*STUB1, ADD1*); GO: endoplasmic reticulum-Golgi intermediate compartment, response to topologically incorrect protein, response to unfolded protein, lysosomal membrane, rough endoplasmic reticulum; KEGG: protein processing in endoplasmic reticulum, lysosome, oxidative phosphorylation, pyruvate metabolism, phagosome; GSEA: up in IA: biological regulation, cellular anatomical entity, cytoplasm, endomembrane system, response to stimulus; up in ctrl: cellular macromolecule metabolic process, cellular metabolic process, gene expression, nuclear lumen, regulation of macromolecule metabolic process; VSMC phenotype in IA: VSCMC-synthesis-phenotype-feature genes
35655614 [82]	GSE75360 GSE122897 GSE13353 GSE166676	PBMC HA: 11 HA, 10 ctrl; tissue: 44 IA, 16 ctrl; 11 RA, 8 UA; AAA tissue sc: 4 AAA, 2 ctrl	mRNA	RNAseq, single-cell RNAseq, microarray	gene expression profiling in IA with HA, identification of potential therapeutic targets	DEGs, monocyte/macrophage-related DEGs from scRNAseq (AAA), TRRUST for TF-gene network, Molecular Complex Detection for subnetworks, GSEA for functional annotation (GO, KEGG)	95 DEGs common for IA and HA including 5 monocyty/macrophage-related DEGs (*IFI30, SERPINE1, HMOX1, IL24, RUNX1*); functional pathways: up: viral protein interaction with cytokine and cytokine receptor, HIF-1 signaling pathway, cytokine–cytokine receptor interaction, receptor ligand activity, phosphatidylinositol-3,4-biphosphate binding; down: exocytic vesicle membrane, synaptic vesicle membrane, neuron to neuron synapse, synaptic membrane, neuronal cell body; TF-gene network: *RUNX1* as hub gene for IA
35918429 [84]	GSE122897 GSE157628 GSE161044	48 IA, 19 ctrl	mRNA	Agilent microarray RNA, RNAseq	gene expression profiling in IA	DEGs, co-expression network with WGCNA modules, clusterProfiler for functional annotation (GO, KEGG), GSVA for pathways, GSEA of the key gene, predicted TFs (Enrichr, hTFtarget), miRNA targets (GeneCards), ferroptosis markers (FerrDb), validation in animal model	DEGs of GSE122897: *CFTR, MTMR7, TSPOAP1, YBX2, ABCC8*; DEGs of GSE157628: *PDE7B, XLOC_011278, LOC100131176, PLEKHA6, GNA14, C12orf40*; WGCNA modules: green (115 hub genes: *ATP1A4, LCNL1, TUB, PPP1R1B, GJB6*); pink (130 hub genes: *KDELR2, SEC24D, FBN1, CALU, COL5A2*); GO-BP: organic acid transport, carboxylic acid transport, organic anion transport, monocarboxylic acid transport, multicellular organismal signaling; KEGG: butanoate metabolism, valine leucine and izoleucine degradation, propanoate metabolism, β alanine metabolism, limonene and pinene degradation; *SLC2A12* as key gene related to the ferroptosis phenotype and ferroptosis marker; TFs: *AR, NANOG*; miRs: mir-223-5p, miR-502-3p
36057911 [85]	GSE122897 GSE75436 GSE15629 GSE75434	72 IA, 36 ctrl	mRNA	Affymetrix microarray RNA, RNAseq	Ferroptosis-related genes’ expression profiling in IA	DEGs, ferroptosis-related genes (FRG), STRING for PPI network, clusterProfiler, MSigDB, GSVA for functional annotation (GO, KEGG, HALLMARKS), GSEA, xCell algorithm for immune cell infiltration, co-expression network for FRG with WGCNA, RegNetwork for predicted miRNAx and TFs, DGIdb for drugs prediction	28 DEFRGs: 22 up, 6 down; PPI network: 17 markers, 7 drivers, 4 suppressors; GO-BP: heme NADPH as acceptor, amino transmembrane acid transporter, single donors molecular incorporation, cyclin-dependent proteine serine-threonine kinase, cytokine receptor factor activator; KEGG: ferroptosis, bladder cancer, HIF-1 signaling pathway, rheumatoid arthritis, IL-17 signaling pathway; HALLMARKS: epithelial–mesenchymal transition, P53 pathway interferone response, apoptosis, oxidative phosphorylation, pancreas β cells; immune response activity in IA: antigen processing and presentation, cytokines, interleukins; immunocyte-FRGs correlation: positive ALOX5-macropahe, negative ATP6V1G2-effector memory CD8 T cell; hub FRGs: *ABCC1, CDKN1A, MT3, ZFP69B*; 2 ferroptosis subtypes; suggested drug targets: *FCGR3A, FCGR2A* (etanercept, rituximab, trastuzumab, cetuximab, infliximab, adalimumab)
28587396 [68]	GSE54083 GSE46337 GSE26969 GSE15629 GSE6551 GSE50867 GSE46336	mRNA: 37 IA, 25 ctrl; miRNA: 11 IA, 7 ctrl	mRNA, miRNA	Agilent, Affymetrix microarray	mRNA and miRNA expression profiling in IA, regulatory networks construction	DEGs, DEmiRNAs, miRNA target prediction (DIANAmT, miRanda, miRDB, miRWalk, PICTAR, TargetScan), GENECODIS for functional annotation (GO, KEGG), miRNA-target gene network construction	15 DEmiRNAs: 10 up (hsa-miR-188-5p, hsa-miR-1183, hsa-miR-18a, hsa-miR-7, hsa-miR-590-5p), 5 down (hsa-miR-425a, hsa-miR-182, hsa-miR-1825, hsa-miR-139-5p, hsa-miR-193b); 1,447 DEGs: 682 up, 765 down; GO-BP: Peptide transport, Amide transport, Positive regulation of phosphorylation, Single-organism catabolic process, Positive regulation of phosphorus metabolic process; GO-CC: Collagen trimer, Endoplasmic reticulum lumen; GO-MF: ATP binding, Adenyl ribonucleotide binding, Adenyl nucleotide binding, ATPase activity coupled to movement of substances, Primary active transmembrane transporter activity; KEGG: Focal adhesion, Pathways in cancer, Cytokine–cytokine receptor interaction, Amoebiasis, Chemokine signaling pathway; key in networks: hsa-miR-7, hsa-miR182, hsa-miR-324-3p, hsa-miR-139-5p, hsa-miR-130b, *RPS6KA3, TSC1, AIM1, GAS7, GFOD1*
35034029 [66]	GSE122897	8 IA, 10 ctrl	mRNA, miRNA	Illumina microarray	miRNA expression profiling and pathways in IA	DEmRNAs, DEmiRNAs, co-expression network with WGCNA, functional annotation (KEGG), miRNA–mRNA regulatory network	955 DEmRNAs: 480 up, 475 down; 46 DEmiRNAs: 36 up, 10 down; KEGG for DEmRNA: ECM–receptor interaction, focal adhesion, cell adhesion molecules (CAMs), complement and coagulation cascades, hematopoietic cell lineage; WGCNA: yellow module, 16 hub miRNAs; KEGG for predicted target DEmRNAs: vascular smooth-muscle contraction, focal adhesion, regulation of actin cytoskeleton, PPAR signaling pathway, calcium signaling pathway
33750300 [64]	GSE66240	6 IA, 12 STA	mRNA, lncRNA, miRNA	RNAseq, miRNA microarray	ceRNA networks in IA	DElncRNAs, DEmiRNAs, DEmRNAs, DAVID for functional annotation (GO, KEGG), miRcode for lncRNA–miRNA interactions, miRDB, miRTArBase, TargetScan for miRNA targets, Cytoscape for ceRNA networks	2914 DEmRNAs: 1807 up, 1107 down; 234 DEmiRNAs: 10 up, 224 down; 341 DElncRNAs: 201 up, 141 down; GO-BP: skeletal system development, positive regulation of cell migration, muscle contraction, inflammatory response, extracellular matrix organization; KEGG: viral myocarditis, vascular smooth-muscle contraction, proteoglycans in cancer, protein digestion and absorption, PI3K-Akt signaling pathway; ceRNA networks with 90 nodes (60 mRNAs, 9 miRNAs, 22 lncRNAs); highest degree: hsa-miR-17, *PVT1, NEAT1, KCNQ1OT1*
35711443 [83]	GSE122897 GSE54083 GSE75436 GSE13353 GSE66239 GSE36791 GSE159610	vessel wall: 39 RA, 29 UA, 28 IA, 51 ctrl; blood: 43 RA, 25 UA, 40 ctrl	mRNA, lncRNA, miRNA	RNAseq, microarray	transcriptomic profiling in IA focused on immune microenvironment	DEGs, DElncRNAs, immune-related DEGs, estimation of immune cell infiltration (single-sample GSEA, ssGSEA), GSEA for functional annotation (GO. KEGG), STRING for PPI network, immunohistochemistry, DEmiRNAs, miRNA targets (miRWalk, lncBase), ceRNA regulatory networks, drug-gene interactions (GDIdb), co-expression network with WGCNA	**IA**: 746 DEmrNAs, 552 DElncRNAs; 1775 immune-related DEGs: 146 up; 99 down; KEGG: JAK-STAT signaling pathway, lysosome, Toll-like receptor signaling pathway, T cell receptor signaling pathway, NOD-like receptor signaling pathway; immune-related DEGs: KEGG up: cytokine–cytokine receptor interaction, viral protein interaction with cyokine and cytokine receptor, Kaposi sarcoma-associated herpesvirus infection, JAK-STAT signaling pathway, chemokine signaling pathway; KEGG down: neuroactive ligand-receptor interaction, axon guidance, Ras signaling pathway, cAMP signaling pathway, MAPK signaling pathway; GO up: positive regulation of T cell activation, lymphocyte proliferation, regulation of T cell activation, leukocyte cell–cell adhesion, positive regulation of peptidyl-tyrosine phosphorylation; GO down: axonogenesis, regulation of neurogenesis, stem cell development, negative regulation of nervous system development, regulation of cell development; infiltrating cells (expression results): **IA**: effector immune cells (macrophage, activated, dendritic cell (DC), natural killer (NK) cell, NK T cell, CD56+ NK cell, myeloid-derived suppressor cell (MDSC), activated CD4 T cell, activated CD8 T cell, γ delta (gd) T cell, regulatory T (Treg) cell, and Type 1 T helper (Th1) cell, **RA vs. UA**: mast cell, neutrophil; PPI network hub genes: *IL6, IL10, STAT1, CXCL10, VEGFA*; WGCNA modules: yellow positively correlated with RA (γ delta T cell, macrophage; enriched in: extracellular matrix organization, external encapsulating structure organization, collagen metabolic process, PI3K-Akt signaling pathway, ECM–receptor interaction), brown negatively correlated with RA (CD56+ NK cell, macrophage; enriched in SMC-contraction-related genes: muscle contraction, regulation of cytosolic calcium ion concentration, calcium ion transmembrane import into the cytosol, cAMP signaling pathway, calcium signaling pathway); potential therapeutics: IL6 inhibitors (Olokizumab, Siltuximab), VEGFA inhibitors (Aflibercept, Bevacizumab, Pegaptanib sodium)

AA, aortic aneurysm; AAA, abdominal aorta aneurysm; AD, aortic dissection; BP, biological process; CC, cellular component; ceRNA, competing endogenous RNA; ctrl, control; DEGs, differentially expressed genes; DElncRNAs, differentially expressed lncRNA; DEmiRNAs, differentially expressed miRNAs; DEmRNAs, differentially expressed mRNAs; ECM, extracellular matrix; EMT, epithelial–mesenchymal transition; ERS, endoplasmic reticulum stress; GO, Gene Ontology; GSEA, Gene Set Enrichment Analysis; HA, hypertension; GSVA, Gene Set Variation Analysis; IA, intracranial aneurysm; IPA, Ingenuity Pathway Analysis; KEGG, Kyoto Encyclopedia of Genes and Genomes; MF, molecular function; MMA, middle meningeal artery; RA, ruptured aneurysm; PPI, protein–protein interaction; RNAseq, RNA sequencing; scRNAseq, single-cell RNA sequencing; STA, superficial temporal artery; TAA, thoracic aortic aneurysm; TAAD, TAA, thoracic aortic aneurysm dissection; TF, transcription factor; UA, unruptured aneurysm; VSMC, vascular smooth-muscle cell; WGCNA, Weighted Gene Co-Expression Network Analysis.

**Table 4:** Studies on RNA expression in blood-related samples in intracranial aneurysm utilizing existing datasets.

PMID/Reference	Datasets ID	Cohorts	Source	RNA Type	Detection/Verification Methods	Aim of the Study	Analytical Methods	Major Findings including Differentially Expressed RNAs, Involved Pathways/Functions (Top 5)
28930970 [89]	GSE36791	43 RA, 18 ctrl	peripheral blood cells	mRNA	Illumina microarray	gene expression profiling in RA and regulatory miRNA prediction	DEGs, DAVID for functional annotation (GO, KEGG), co-expression network with WGCNA modules, cGRNB for predicted miRNA–gene interactions	304 DEGs: 167 up, 137 down; GO down: translational elongation, structural constituent of ribosome, cytosolic ribosome, ribosomal subunit, ribosome; KEGG down: Ribosome; WGCNA modules: up (GO, KEGG): blue (cell fraction, IgG binding), brown (nucleosome assembly, chromatin assembly), turquoise (innate immune response, inflammatory response), yellow (interleukin-1 receptor activity, interleukin-1 binding); down: blue (cytolysis, cellular defense response), turquoise (translational elongation, Ribosome); 16 predicted regulatory miRNAs (hsa-miR-1304, hsa-miR-373 hsa-miR-514, hsa-miR-33b, hsa-miR-568)
31026661 [90]	GSE36791	43 RA, 18 ctrl	peripheral blood cells	mRNA	Illumina microarray	gene expression profiling in RA	DEGs, DAVID for functional annotation (GO, KEGG), STRING for PPI network, GSEA for pathways of key genes	528 DEGs: 311 up (*C19ORF59, CA1, IL1R2, ARG1, ANXA3*), 217 down (*MAL, CD7, ABLIM1, CD6, IL2RB*); GO: translation, T cell activation, innate immune response, immunoglobulin-mediated immune response, protein phosphorylation; KEGG: Ribosome, Hematopoietic cell lineage, Transcriptional misregulation in cancer, T cell receptor signaling pathway, Systemic lupus erythematosus; PPI network with hub genes (*ARG1, MAPK14, RPS2, SPI1, FYN*); GSEA (potential aSAH biomarkers): up: *MAPK14, CEBPB, FLOT1*, down: *CD4*
32084215 [86]	GSE36791 GSE73378	146 RA, 125 ctrl; validation in: 10 UA, 10 RA, 10 ctrl	peripheral blood cells	mRNA	Illumina microarray; validation: qPCR	gene expression profiling and hub genes in RA	DEGs, co-expression network with WGCNA modules and hub genes, clusterProfiler for functional annotation (GO, KEGG)	396 DEGs (*BASP1, CD74, CEBPB, ECHDC2, GZMK*); WGCNA modules: turquoise (190 hub genes), blue (38 hub genes), brown (10 hub genes); GO-BP: rRNA processing, Ribonucleoprotein complex biogenesis, rRNA metabolic process, Ribosome biogenesis, ncRNA processing; GO-CC: Cytosolic ribosome, Ribosomal subunit, Ribosome, Focal adhesion, Cell–substrate adherens junction; GO-MF: Structural constituent of ribosome; KEGG: Ribosome; 6 potential biomarkers of the progression and IA rupture: *BASP1, CEBPB, ECHDC2, GZMK, KLHL3, SLC2A3*
33174039 [92]	GSE36791	43 RA, 18 ctrl	peripheral blood cells	mRNA	Illumina microarray	gene expression profiling in RA, identification of aSAH-related lncRNA	DEGs, DElncRNA, co-expression network with WGCNA modules, DAVID for functional annotation (GO, KEGG), lncRNA–mRNA regulatory network construction (Cytoscape), Comparative Toxigenomics Database (DTB) for aSAH-related pathways	25 DElncRNAs: 12 up, 13 down; 1979 DEGs: 781 up, 1198 down; WGCNA modules (DEGs and/or DElncRNAs): purple (50), turquoise (201), green (140), pink (76); ceRNAs networks with 382 nodes, 7 up lncRNA (HCG27, ZFAS1 antisense RNA, LINC002665, MRV1-AS1, CYP1B1-AS1); GO-BP in WGCNA modules: green: leukocyte activation, inflammatory response, response to wounding, cell activation, positive regulation of apoptosis; pink: intracellular signaling cascade, phosphate metabolic process, phosphorus metabolic process, phosphorylation, protein amino acid phosphorylation; purple: regulation of apoptosis, regulation of programmed cell death, regulation of cell death, apoptosis, programmed cell death; turquoise: carbohydrate catabolic process, cellular carbohydrate catabolic process, defense response, hexose catabolic process; KEGG: Chemokine signaling pathway, Cytokine–cytokine receptor interaction, MAPK signaling pathway, Leukocyte transendothelial migration, Toll-like receptor signaling pathway
33567366 [87]	GSE36791 GSE54083 GSE13353 GSE26969 GSE122897	internal validation: 62 RA, 16 UA, 31 ctrl; external validation: 22 RA, 21 UA, 16 ctrl	peripheral blood	mRNA	microarray	gene expression profiling in iA, predictive models for aSAH	DEGs, co-expression network with WGCNA modules, clusterProfiler, GSEA for functional annotation (GO, KEGG), STRING for PPI network and hub genes (CytoHubba), prediction model construction (LASSO)	433 DEGs (up: *CEBPD, MMP9, IL18RAP, IL1R2, S100A12*; down: *CEACAM8, EME2, ADAMTS10, XK, ARG1*); WGCN modules: black with strongest association with RA; GO-BP: inflammatory response, defense response to bacterium, innate immunity response, positive regulation of mast cell degranulation, MyD88-dependent toll-like receptor signaling pathway; GO-CC: extracellular exosome, extracellular space, specific granule, plasma membrane, IPAF inflammasome complex; GO-MF: catalytic activity, glucose binding, cysteine-type endopeptidase inhibitor activity, protein homodimerization activity, transcription corepressor activity; KEGG: inflammatory bowel disease (IBD), amoebiasis, legionellosis, fatty acid biosynthesis, salmonella infection; PPI network with 30 hub genes; 4 rupture-related genes: *TNFAIP6, NCF2, OSM, IRAK3*
34485395 [88]	GSE36791 GSE6551	51 RA, 6 UA, 18 ctr	peripheral blood cells	mRNA	Illumina microarray/qPCR	gene expression profiling in RA	DEGs, clusterProfiler for functional annotation (GO, KEGG), CIBERSORT for cell composition analysis, STRING for PPI network, MCODE for subnetworks	**RA vs. ctrl**: 58 DEGs:50 up, 8 down; GO-BP: neutrophil activation, neutrophil degranulation, neutrophil activation involved in immune response, neutrophil-mediated immunity, killing of cells of other organism; KEGG: Staphylococcus aureus infection, Transcriptional misregulation in cancer, Viral protein interaction with cytokine and cytokine receptor, Cytokine–cytokine receptor interaction, Inflammatory bowel disease (IBD); CIBERSORT: B_cells_memory, T_cells_CD8, T_cells_CD4_memory_resting, T_cells_CD4_naive, Macrophages_M0, Macrophages_M2, NK_cells_resting, monocytes, neutrophils; PPI network with 24 hub genes: *IL2RB* and *CCR7*—down in RA
34542421 [94]	GSE36791	43 RA, 18 ctrl	peripheral blood cells	mRNA	Illumina microarray/qPCR	gene expression profiling in RA	co-expression network with WGCNA modules and hub genes, METASCAPE for functional annotation (GO, KEGG)	WGCNA modules (hub genes): red (*ARRB2, CSF3R, DENND3, DYSF, GMIP*); blue (*ABCF1, ABHD14A, ACSL1, ADA, AIP*); brown (*ABHD14A, ACAD9, ACTR5, AFG3L2, ALKBH3*); cyan (*ACTR1A, AKAP11, API5, BRIX1, BUB3*); GO-BP: peptide biosynthetic process, rRNA processing, ncRNA processing, cotranslational protein targeting to membrane, SRP-dependent cotranslational protein targeting to membrane; KEGG: HTLV-1 infection, Toxoplasmosis, RNA transport, Th17 cell differentiation, spliceosome; 7 genes as potential aSAH biomarkers: *CD27, ANXA3, ACSL1, PGLYRP1, ALPL, ARG1, TPST1*; 3 genes changed with aSAH progression: *ANXA3, ALPL, ARG1*
32756097 [91]	GSE50867	40 IA, 20 ctrl	plasma	circulating miRNA	Agilent microarray	circulating miRNA expression profiling in IA	co-expression network with WGCNA modules and hub genes, GSVA (hub miRNAs—disease state), predicted targets (diana_microt, elmmo, microcosm, Miranda, mirdb, pictar, pita, TargetScan), STRING for PPI network, Cytoscape for miRNA–mRNA network; clusterProfiler for functional annotation (GO, KEGG)	WGCNA brown module: GO-BP: gland development, cell cycle G1/S phase transition, positive regulation of cell cycle, cell–cell adhesion via plasma-membrane adhesion molecules, G1/S transition of mitotic cell cycle; GO-MF: DNA-binding transcription activator activity RNA polymerase II-specific, proximal promoter sequence-specific DNA binding, RNA polymerase II proximal promoter sequence-specific DNA binding, SMAD binding; WGCNA modules (hub miRNAs): brown (hsa-miR-363-3p, hsa-miR-192-5p, hsa-miR-425-5p, hsa-miR-25-3p, hsa-miR-423-5p), green (hsa-miR-1281, hsa-miR-1825, hsa-miR-498-5p, hsa-miR-1280, hsa-miR-1234-3p); miRNA–mRNA network: 243 nodes (*PTEN, VEGFA, CCND1, MDM2, CREB1*) and hub miRNA (hsa-miR-93-5p); key pathway: PI3K/Akt signaling pathway
33990177 [93]	GSE36791	43 RA, 18 ctrl	peripheral blood cells	mRNA, lncRNA	Illiumina microarray	mRNA and lncRNA expression profiling in RA	DEGs, DElncRNAs, DAVID for functional annotation (GO, KEGG), co-expression network with WGCNA modules, lncRNA–mRNA regulatory network construction (Cytoscape), GSEA in regulatory network	25 DElncRNAs: 10 up (MRVI1-AS1, ZFAS1, FAM157C, CYP1B1-AS1, LINC02035); 15 down (INTS6-AS1, SNHG5, SNHG14, PRKCQ-AS1, DANCR), 536DEmRNAs: 307 up (*S100A12, HP, IL18R1, CST7, MMP9*); 229 down (*FCER1A, CLC, CD27, IL2RB, CCR7*); GO-BP: regulation of lymphocyte activation, positive regulation of cell activation, regulation of cell activation, positive regulation of immune response, positive regulation of response to stimulus; KEGG: adipocytokine signaling pathway, T cell receptor signaling pathway, NOD-like receptor signaling pathway, cytokine–cytokine receptor interaction, ribosome; WGCNA modules (hub genes): yellow (*CASP4, TNFSF13B, FNDC3B, N4BP2L2, OSM*), blue (*HIST2H2AB, ATP6V1C1, NFE2, USB1, NTN3*), red (*CEACAM4, SMAP2, CSGALNACT2, TLR2, TMIGD3*), brown (*IDI1, GNAI3, E2F3, WSB1, NRBF2*), black (*PFKFB4, SLC9A8, LIN7A, MGAM2, LILRB3*), pink (*ENTPD1, USP32, LTB4R, FGR, SBNO2*); regulatory network: LINC00265 (*NFKBIA, IRAK3*), LINC00937 (*NFKBIA*)

aSAH, aneurysmal subarachnoid hemorrhage; ctrl, control; BP, biological process; CC, cellular component; DEGs, differentially expressed genes; DElncRNAs, differentially expressed lncRNA; GO, Gene Ontology; GSEA, Gene Set Enrichment Analysis; GSVA, Gene Set Variation Analysis; IA, intracranial aneurysm; KEGG, Kyoto Encyclopedia of Genes and Genomes; MF, molecular function; RA, ruptured aneurysm; PPI, protein–protein interaction; qPCR, quantitative PCR; UA, unruptured aneurysm; WGCNA, Weighted Gene Co-Expression Network Analysis. In some studies, GO and KEGG terms were not analyzed separately for up- and down-regulated DERNAs but only for DERNAs as a whole. Data presented in the table reflect available data.

## Data Availability

Data sharing not applicable.
